# Has Omicron Changed the Evolution of the Pandemic?

**DOI:** 10.2196/35763

**Published:** 2022-01-31

**Authors:** Alexander L Lundberg, Ramon Lorenzo-Redondo, Egon A Ozer, Claudia A Hawkins, Judd F Hultquist, Sarah B Welch, PV Vara Prasad, James F Oehmke, Chad J Achenbach, Robert L Murphy, Janine I White, Robert J Havey, Lori Ann Post

**Affiliations:** 1 Buehler Center for Health Policy and Economics Robert J. Havey, MD Institute for Global Health Northwestern University Chicago, IL United States; 2 Department of Emergency Medicine Feinberg School of Medicine Northwestern University Chicago, IL United States; 3 Department of Medicine, Division of Infectious Diseases Feinberg School of Medicine Northwestern University Chicago, IL United States; 4 Center for Pathogen Genomics and Microbial Evolution Robert J. Havey, MD Institute for Global Health Northwestern University Chicago, IL United States; 5 Center for Global Communicable and Emerging Infectious Diseases Robert J. Havey, MD Institute for Global Health Northwestern University Chicago, IL United States; 6 Sustainable Intensification Innovation Lab Kansas State University Manhattan, KS United States; 7 Robert J. Havey, MD Institute for Global Health Feinberg School of Medicine Northwestern University Chicago, IL United States; 8 Department of Medicine, General Internal Medicine and Geriatrics Feinberg School of Medicine Northwestern University Chicago, IL United States

**Keywords:** Omicron, SARS-CoV-2, public health surveillance, VOC, variant of concern, Delta, Beta, COVID-19, sub-Saharan Africa, public health, pandemic, epidemiology

## Abstract

**Background:**

Variants of the SARS-CoV-2 virus carry differential risks to public health. The Omicron (B.1.1.529) variant, first identified in Botswana on November 11, 2021, has spread globally faster than any previous variant of concern. Understanding the transmissibility of Omicron is vital in the development of public health policy.

**Objective:**

The aim of this study is to compare SARS-CoV-2 outbreaks driven by Omicron to those driven by prior variants of concern in terms of both the speed and magnitude of an outbreak.

**Methods:**

We analyzed trends in outbreaks by variant of concern with validated surveillance metrics in several southern African countries. The region offers an ideal setting for a natural experiment given that most outbreaks thus far have been driven primarily by a single variant at a time. With a daily longitudinal data set of new infections, total vaccinations, and cumulative infections in countries in sub-Saharan Africa, we estimated how the emergence of Omicron has altered the trajectory of SARS-CoV-2 outbreaks. We used the Arellano-Bond method to estimate regression coefficients from a dynamic panel model, in which new infections are a function of infections yesterday and last week. We controlled for vaccinations and prior infections in the population. To test whether Omicron has changed the average trajectory of a SARS-CoV-2 outbreak, we included an interaction between an indicator variable for the emergence of Omicron and lagged infections.

**Results:**

The observed Omicron outbreaks in this study reach the outbreak threshold within 5-10 days after first detection, whereas other variants of concern have taken at least 14 days and up to as many as 35 days. The Omicron outbreaks also reach peak rates of new cases that are roughly 1.5-2 times those of prior variants of concern. Dynamic panel regression estimates confirm Omicron has created a statistically significant shift in viral spread.

**Conclusions:**

The transmissibility of Omicron is markedly higher than prior variants of concern. At the population level, the Omicron outbreaks occurred more quickly and with larger magnitude, despite substantial increases in vaccinations and prior infections, which should have otherwise reduced susceptibility to new infections. Unless public health policies are substantially altered, Omicron outbreaks in other countries are likely to occur with little warning.

## Introduction

### Background

The Omicron (B.1.1.529) variant was identified in Botswana on November 11, 2021 [1]. This novel variant has an unprecedented average of 50 mutations, including around 30 mutations in the spike protein. In vitro studies and epidemiological surveys suggest that Omicron is able to spread more rapidly, but more information is needed to define transmission rates, determine if it evades vaccine-elicited or natural immunity, and determine if it influences disease severity or pathogenesis [2]. More data are needed to fill in these critical knowledge gaps to best inform public health practices as Omicron continues to spread [3].

### Omicron Compared to Other Variants

After the first cases of Omicron were identified in Botswana, it first spread to several countries in sub-Saharan Africa (SSA) and has since spread globally [4]. Since then, Omicron has been identified in more than 140 countries [5,6]. Preliminary investigations estimate that Omicron may have infected 3-6 times as many people as the Delta variant over this same time period [3,7-10]. Given that most outbreaks of new variants have occurred during periods of low incidence, it is hard to estimate how Omicron will behave in competition with other variants in regions of high incidence. Preliminary data from Europe suggest Omicron may outcompete Delta, though it is unclear if these variants are targeting the same population [11]. Specifically, it is not clear if vaccinations or prior infections impact the infectivity and/or transmissibility of Omicron to the same extent as other variants. Although viral reproduction rates may provide some proxy of transmission risk, no studies have yet been completed that stratify Omicron’s risk in different populations. To that end, this study employs surveillance data and empirically tested transmission metrics in the first countries to experience outbreaks in SSA to determine how Omicron compares to the first wave of SARS-CoV-2 virus as well as its subsequent variants of concern, including Alpha, Beta, and Delta.

### Variants of Concern

Since late 2020, variants of the SARS-CoV-2 virus that pose increased risks have been identified, named, and monitored [12]. A variant that poses increased risk to human health is classified as a variant of interest (VOI) if it has genetic changes that affect transmission, severity, immune system protection, or treatment effectiveness and is associated with community spread [12]. VOIs that result in an increase in transmissibility or disease severity, or that are not controlled through public health, vaccination, or medical therapy interventions, are designated as variants of concern (VOC) [12]. Since May 2021, VOIs and VOCs have been named by the World Health Organization using the Greek alphabet. One VOC, Beta, originated in SSA, with the earliest documented sample in South Africa in May 2020. VOC designation was not declared for the Beta variant until December 18, 2020 [12]. The most recent VOC, Omicron, was identified in multiple locations in November 2021 and was officially designated a VOC on November 26, 2021 [12].

### Omicron in Southern African Countries

Since its debut more than 2 months ago, Omicron has been sequenced all over the world and appears to be responsible for driving several outbreaks of SARS-CoV-2 or causing existing outbreaks to accelerate [13,14]. Because the acceleration of daily transmissions has often been driven by multiple variants within a given country’s outbreak, such as in the case of the United States or the United Kingdom [15-17], it is difficult to disentangle the individual burden each variant places on a given population [18].

On November 11, 2021, the date that Omicron was first sequenced in Botswana, the rate of new SARS-CoV-2 transmissions in the United Kingdom was 34,427 cases per day. Estimates are based on a 7-day moving average, or a rate of 50.47 daily new cases per 100,000 population [4]. This transmission rate is more than 5 times that of an outbreak; the Centers for Disease Control and Prevention (CDC) defines an outbreak as 10 daily new cases per 100,000 population. Omicron was identified in the United Kingdom while they were already in the middle of an outbreak that involved other VOCs. Conversely, with the exception of Botswana, none of the countries in the south of SSA were in an outbreak. In fact, the average daily speed for SSA or daily new transmissions was 0.17 per 100,000 population, and that rate was decelerating by 0.08 cases per day during the week that Omicron was first sequenced in Botswana. This is consistent with previously reported outbreaks in most SSA countries, which occurred at periods of low incidence and thus were driven largely by one variant at a time. Therefore, SSA countries provide an opportunity to understand how Omicron affects an entire population compared to other VOCs without having to differentiate the involvement of other variants within an outbreak.

### Objective

The objective of this study is to examine the status of the SARS-CoV-2 pandemic in SSA and to model novel transmission metrics to determine if Omicron is more transmissible than other VOCs.

## Methods

### Novel Surveillance Metrics

This report will present both standard and new validated surveillance and transmission metrics [19-29] on the status of the SARS-CoV-2 pandemic in SSA countries over the past 3 weeks. The Foundation for Innovative New Diagnostics [30] compiles data from multiple sources across individual websites, statistical reports, and press releases; data for the most recent 8 weeks were accessed from the GitHub repository [31]. This produced a panel of 47 countries with 120 days for each country (n=5640). An empirical difference equation was specified in which the number of positive cases in each country at each day is a function of the prior number of cases and weekly shift variables that measure whether the contagion was growing faster/slower/at the same rate compared to the previous weeks. The dynamic panel model was estimated using the generalized method of moments approach by implementing the Arellano-Bond estimator in R (version 4.1.1; R Foundation for Statistical Computing) with the *plm* package (version 2.4-1) [32,33].

Arellano-Bond estimation of difference equations has several statistical advantages over R naught [26,28,34-38]: (1) it allows for statistical examination of the model’s predictive ability and the validity of the model specification, (2) it corrects for autocorrelation and heteroscedasticity, (3) it has good properties for data with a small number of time periods and large number of countries, and (4) it corrects for omitted variable issues and provides a statistical test of correction validity. With these advantages, the method is applicable to ascertain and statistically validate changes in the evolution of the pandemic within a period of a week or less, including changes in the reproduction rate [27]. Empirically, we validated this technique based on the predictive ability of past data that resulted in the derivation of speed, acceleration, jerk, and 7-day persistence, which follow the definitions and methods described by Oehmke and colleagues [27,34].

Traditional surveillance indicators include total cases and deaths, 7-day moving average of new cases, and 7-day moving average of deaths. Enhanced surveillance metrics [26-28] include the following: (1) speed (the weekly average number of new positive tests per day divided by the total country population and multiplied by 100,000), (2) acceleration (the weekly average of the day-over-day change in the speed of infection), (3) jerk (the week-over-week change in the acceleration rate of transmissions), and (4) seven-day persistence effect on speed, which refers to the number of new cases reported today that are statistically attributable to new cases reported 7 days ago. We measure the transmission inflation factor by dividing the rate of new cases each week by the rate of new cases on December 3 [4]. Although standard surveillance metrics identify the presence and severity of an outbreak, they do not explain whether an outbreak is contracting, escalating, or imminent. Our additional transmission metrics do.

We also used an extension of the sample to examine how Omicron may have shifted the evolution of the pandemic. To compare Omicron outbreaks to those caused by earlier VOCs, we also included Arellano-Bond estimates for the entire year of 2021. We controlled for cumulative vaccinations and infections because SSA countries had far fewer vaccinations and infections at the time of earlier outbreaks compared to the current Omicron outbreaks. An interaction term between an indicator for the emergence of Omicron with the 1- and 7-day lags of cases provides a test for whether Omicron has shifted the nature of persistence in the pandemic.

### Publicly Available Molecular Data

Data on the number of sequenced variants over time per country were obtained from publicly available sequences in Global Initiative on Sharing Avian Influenza Data (GISAID) [39]. We collected clade designations from sequences using Nextclade nomenclature [40] and lineage designations using Pangolin nomenclature for SARS-CoV-2 [41,42]. Additionally, we contrasted prevalence data with the compiled data available in outbreak.info [43].

### Modeling

We first plotted the spike in cases by variant and country. To compare trends in the rate of new infections under each variant, we standardized the point at which a country eclipses the CDC threshold of an outbreak as day 0. Within each country, we followed the rate of SARS-CoV-2 infections in the 4 weeks up to an outbreak threshold and in the 4 weeks afterward, subject to the limits of the most recent available data. This standardization allows for a comparison of the speed, acceleration, and magnitude of the Omicron outbreaks relative to those driven by earlier VOCs.

Because these trend comparisons do not control for differences in population vaccinations and prior infections during the various outbreaks, we added these mediators as control variables in a dynamic panel regression. We modeled the rate of new infections as a function of infections on the previous day and previous week. These lagged infection rates measure persistence in the pandemic, or the extent to which cases today echo forward into tomorrow and next week.

The model contains an indicator variable equal to 1 if the calendar date was on or after November 1, 2021, and equal to 0 if the calendar date was earlier. This indicator is meant to capture a conservative estimate of the time window in which the Omicron variant originated. Interactions between this indicator and infections on the previous day and week provide a test for whether Omicron shifted the trajectory of the pandemic. The model also controlled for cumulative vaccinations and infections in the country population, and included an indicator for weekend dates, which are subject to spotty data reports.

We used the Arellano-Bond estimator to generate coefficient estimates, along with their standard errors, for a sample period covering January 1, 2021, through December 31, 2021. This time period covers the recent Omicron outbreaks as well as outbreaks driven by other VOCs.

## Results

Table 1 provides standard surveillance metrics along with our novel metrics of transmission for 7 data points between December 3, 2021, and January 17, 2022. For a complete surveillance and transmission table that includes daily figures for all SSA countries over the past 7 weeks, current through January 17, 2021, please refer to Multimedia Appendix 1 [4].

**Figure 1:**
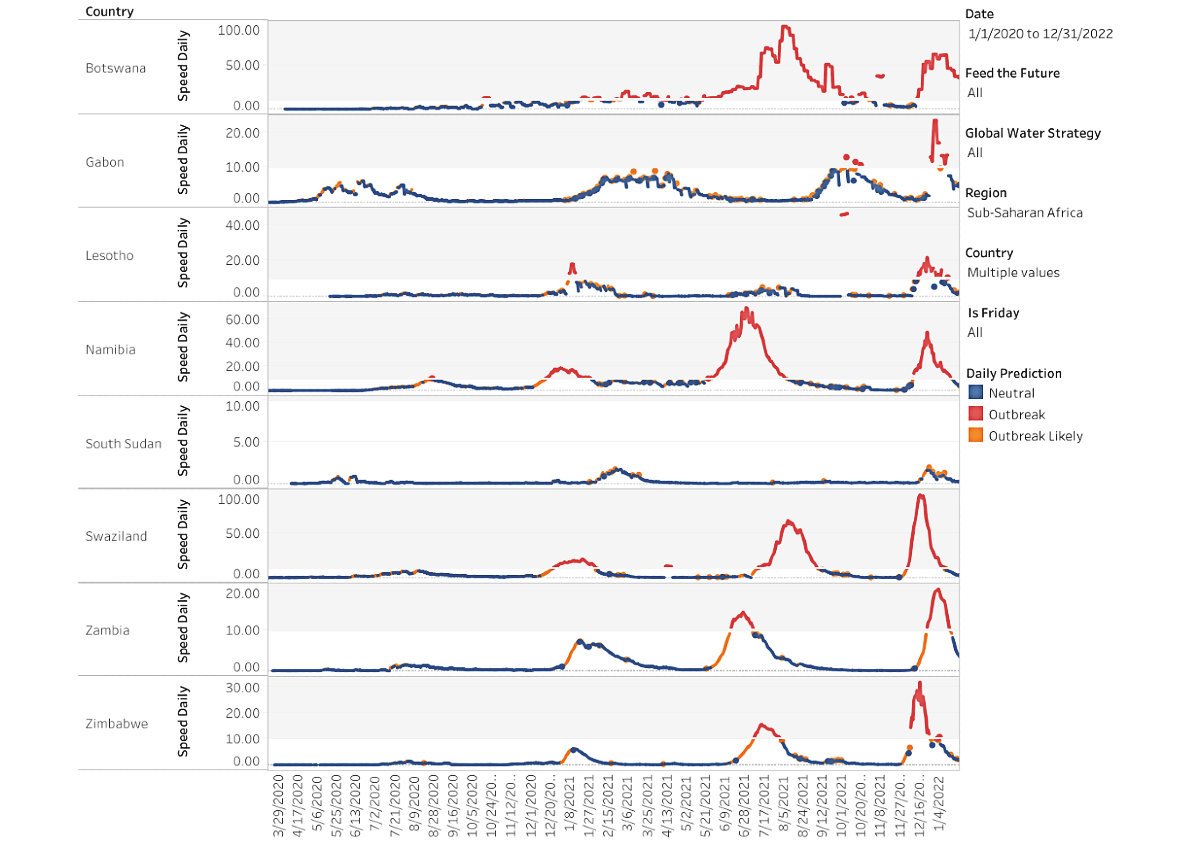
First sub-Saharan countries to experience an Omicron outbreak.

The daily speed of the pandemic is defined as the number of new cases per day per 100,000 population. If we use the CDC threshold for an outbreak or a threshold of 10 daily new cases per 100,000 population, these eight countries in our study group were in an outbreak sometime between December 3, 2021, and January 17, 2022: Botswana, Eswatini (formerly known as Swaziland), Gabon, Lesotho, Namibia, South Africa, Zambia, and Zimbabwe (Figure 1) [4]. In the truncated Table 1, we excluded Cabo Verde, Comoros, and Seychelles because outbreaks in small densely populated island nations included other VOCs when Omicron entered into the equation. Although Zambia, Namibia, and Botswana currently remain in an outbreak (with a daily rate of new cases per 100,000 population of 11.6, 13.5, and 46.6, respectively), Gabon, Lesotho, South Africa, Eswatini, and Zimbabwe’s outbreaks have ended and continue to cycle down at a daily rate of 7.6, 6.3, 9.1, 7.4, and 4.1, respectively.

To put the outbreaks in perspective, we standardized the data using December 3, 2021, as the base rate of daily cases. The inflation factor is the rate of new cases on any given day divided by the base rate on December 3, 2021. By early December, Omicron was driving the escalation of new cases in sub-Saharan countries. Eswatini and South Africa’s Omicron escalation of cases were first to develop and they are the only two countries whose baselines have returned to their December 3, 2021, baselines. The rate of new cases increased for Botswana, Gabon, Lesotho, Namibia, South Africa, Eswatini, Zambia, and Zimbabwe at their apex in the outbreak by 26.8-fold, 22.4-fold, 39.7-fold, 24.9-fold, 3.4-fold, 12.2-fold, 176-fold, and 31.9-fold, respectively.

Our novel transmission metric, 7-day persistence, is based on a 120-day panel of data and measures the number of new cases per day per 100,000 that are a function of novel infections 7 days earlier. Essentially, it measures how outbreaks and transmissions echo forward. Measuring the persistence rate of SARS-CoV-2 avoids the limitations and data bias in the measurement of R naught, such as sampling error and missing data [26,27]. It is the echoing forward of transmissions that explains the underlying condition that causes a clustering of new cases. Persistence is a transmission metric that is the first to signal a potential outbreak because it is based on 120 days of data versus the 7 days used in standard surveillance. As an example, Botswana showed an upward trend in persistence 2 weeks before its Omicron outbreak had reached the CDC threshold of 10 cases per 100,000 population (Table 1). The increase in speed itself only became evident about 7 days before the threshold was reached. Persistence continues to increase for an additional week after the apex of the outbreak as it is the echo forward of cases.

Our model also calculates the weekly rate of acceleration and jerk. The acceleration rate helps to identify countries that are at the beginning, middle, or end of an outbreak, even if a country still has relatively few new SARS-CoV-2 cases per day. In addition, these transmission metrics can inform when a spike in cases is still accelerating and at risk for exponential growth or when an outbreak is slowing, reaching its apex, or decelerating from day to day, whereas the jerk measures shifts in the rates of acceleration week over week.

Exponential growth measures the expansion and contraction of the outbreak. At their zenith, Eswatini and Namibia had the largest weeks of exponential growth at 454.8 and 268.1, respectively.

**Table 1:** Standard and novel surveillance metrics for the first countries to experience an Omicron-only outbreak, December 3, 2021-January 17, 2022.

Country and date		New cases 7-day moving average	New cases/100,000 7-day moving average	Inflation factor	7-day persistence	Acceleration weekly	Jerk weekly	Exponential growth potential weekly
**Botswana**								
	3-Dec-2021	56	2.3	1.0	1.0	–3.0	8.7	–7.0
	10-Dec-2021	113	4.7	2.0	3.9	16.5	19.4	23.3
	17-Dec-2021	539	22.5	9.6	5.6	124.6	108.1	140.0
	24-Dec-2021	1304	54.4	23.2	30.0	223.4	98.9	291.7
	31-Dec-2021	1502	62.7	26.8	73.2	57.8	–165.6	159.2
	7-Jan-2022	1478	61.7	26.3	85.2	–7.0	–64.8	–55.2
	14-Jan-2022	1118	46.6	19.9	30.4	–105.2	–98.2	–185.3
**Gabon**								
	3-Dec-2021	24	1.1	1.0	0.6	–4.9	–1.6	–6.0
	10-Dec-2021	23	1.0	1.0	1.7	–0.4	4.6	–1.6
	17-Dec-2021	27	1.2	1.1	1.2	1.4	1.8	3.4
	24-Dec-2021	42	1.9	1.8	1.6	4.6	3.2	7.7
	31-Dec-2021	537	23.6	22.4	2.5	152.0	147.4	158.3
	7-Jan-2022	306	13.4	12.7	32.2	–71.0	–223.0	–81.7
	14-Jan-2022	173	7.6	7.2	6.6	–40.7	30.3	–46.6
**Lesotho**								
	3-Dec-2021	12	0.5	1.0	0.1	2.5	2.9	3.1
	10-Dec-2021	87	4.0	7.3	0.9	24.4	21.9	26.3
	17-Dec-2021	264	12.2	22.3	4.7	57.4	33.0	70.2
	24-Dec-2021	471	21.8	39.7	16.1	67.0	9.6	101.2
	31-Dec-2021	116	5.4	9.8	29.0	–115.0	–182.1	–65.8
	7-Jan-2022	320	14.8	27.0	7.2	66.2	181.3	83.0
	14-Jan-2022	136	6.3	11.5	7.2	–59.7	–126.0	–51.4
**Namibia**								
	3-Dec-2021	51	2.0	1.0	0.1	10.9	9.5	12.3
	10-Dec-2021	246	9.5	4.8	3.3	52.8	41.9	59.3
	17-Dec-2021	495	19.1	9.7	11.2	67.3	14.6	94.9
	24-Dec-2021	1269	49.0	24.9	25.5	209.4	142.1	268.1
	31-Dec-2021	628	24.3	12.3	65.9	–173.3	–382.7	–171.6
	7-Jan-2022	506	19.6	9.9	33.0	–33.0	140.3	–67.3
	14-Jan-2022	350	13.5	6.9	9.6	–42.2	–9.1	–63.2
**South Africa**								
	3-Dec-2021	6982	11.6	1.0	2.2	67.3	58.1	74.0
	10-Dec-2021	15,467	25.8	2.2	19.1	98.9	31.6	133.6
	17-Dec-2021	23,437	39.0	3.4	30.2	92.9	–6.0	159.3
	24-Dec-2021	16,654	27.7	2.4	51.7	–79.1	–172.0	–123.9
	31-Dec-2021	9311	15.5	1.3	37.0	–85.6	–6.5	–96.4
	7-Jan-2022	7932	13.2	1.1	20.9	–16.1	69.5	–38.6
	14-Jan-2022	5461	9.1	0.8	6.5	–28.8	–12.7	–42.8
**Eswatini**								
	3-Dec-2021	91	7.7	1.0	0.2	50.8	49.0	52.4
	10-Dec-2021	573	48.9	6.3	12.7	288.3	237.6	314.2
	17-Dec-2021	1101	93.9	12.2	57.3	314.7	26.4	454.8
	24-Dec-2021	725	61.9	8.0	124.1	–224.1	–538.8	–311.5
	31-Dec-2021	308	26.3	3.4	82.5	–249.2	–25.1	–214.1
	7-Jan-2022	152	12.9	1.7	35.4	–93.3	155.8	–91.9
	14-Jan-2022	87	7.4	1.0	6.3	–38.9	54.4	–44.8
**Zambia**								
	3-Dec-2021	21	0.1	1.0	0.0	0.4	0.4	0.5
	10-Dec-2021	66	0.3	3.2	0.2	1.7	1.3	2.0
	17-Dec-2021	530	2.8	25.8	0.4	17.2	15.5	18.3
	24-Dec-2021	2071	10.9	100.7	3.8	57.0	39.9	66.1
	31-Dec-2021	3620	19.1	176.0	14.9	57.3	0.3	87.6
	7-Jan-2022	3429	18.1	166.7	26.3	–7.1	–64.4	–30.0
	14-Jan-2022	2203	11.6	107.1	9.0	–45.4	–38.3	–60.8
**Zimbabwe**								
	3-Dec-2021	515	3.4	1.0	0.1	22.3	22.4	23.1
	10-Dec-2021	2625	17.4	5.1	5.6	97.9	75.6	109.2
	17-Dec-2021	4821	31.9	9.4	20.5	101.9	4.0	150.9
	24-Dec-2021	1881	12.5	3.7	42.4	–136.4	–238.2	–109.1
	31-Dec-2021	1503	10.0	2.9	16.7	–17.5	118.8	–35.0
	7-Jan-2022	1146	7.6	2.2	13.5	–16.6	1.0	–29.7
	14-Jan-2022	622	4.1	1.2	3.7	–24.3	–7.8	–26.5

### How Do Omicron Outbreaks Compare to Other VOC Outbreaks?

We examined the daily speed of the pandemic or the number of daily new cases per 100,000 population and found that Gabon, Lesotho, South Africa, Eswatini, Zambia, and Zimbabwe have already set new 2-year outbreak records in the first 4 weeks of the outbreak, in December 2021. All the countries in Figure 1 whose current outbreaks are driven by Omicron, except Botswana, had more new daily infections than during every other outbreak caused by either the original SARS-CoV-2 variant, Beta, or Delta. Most of these countries recorded peak speeds during the outbreak of the Delta variant; however, the exponential growth of Omicron cases has reversed course and contracted. It is remarkable that not only did Omicron result in record highs, but also those highs took fewer days to reach.

For some of the countries currently in an outbreak, such as Botswana and Zambia, the rate of increase is slowing, which means even though these countries are in an outbreak, the increase in daily new cases slowed between the last week in December and the first week in January 2022, compared to the prior 4 weeks. In contrast, sub-Saharan countries where Omicron began transmitting and escalating later are not slowing (Multimedia Appendix 1). Not only did the 7-day moving average, rate of new cases, and daily new cases increase more rapidly with Omicron than with other VOCs, but the rate itself increased when compared to the prior 2-4 weeks.

**Figure 2:**
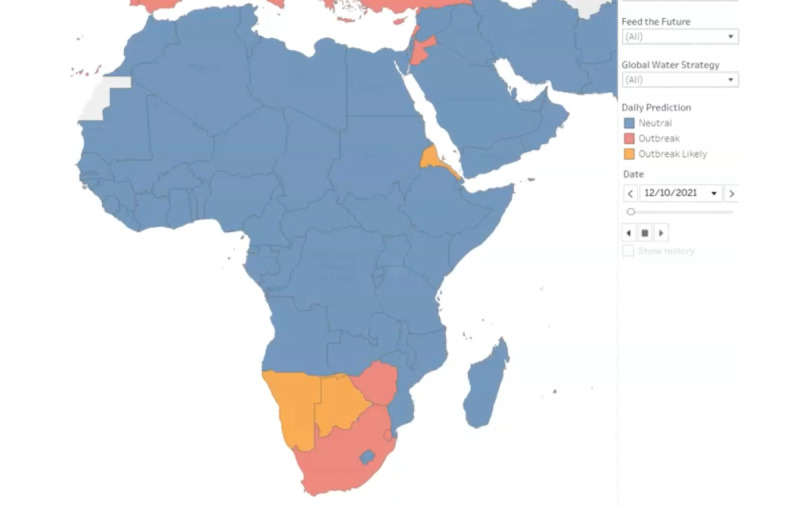
A screen capture of a dynamic map of transmission of the SARS-CoV-2 pandemic through Africa after the Omicron variant was identified. Full video can be viewed here: [44].

Figure 2 demonstrates how the SARS-CoV-2 pandemic transmitted through Africa after the Omicron variant was identified. Countries that light up as orange have been experiencing a surge in cases for 7 consecutive days. Blue countries indicate neutral growth for 24 hours and countries in red exceed the outbreak threshold of 10 daily new cases per 100,000 population. Since November 11, 2021, a number of countries in Africa had outbreaks forming, especially those countries in the south of the continent [4]. The figures for each country, presented below, differentiate between the VOCs.

The evolution of Omicron in the first 8 African countries to experience an Omicron-only outbreak—Botswana, Eswatini, Gabon, Lesotho, Namibia, South Africa, Zambia, and Zimbabwe—are depicted in the figures presented below. To compare the evolution of infections to outbreaks driven by earlier VOCs, a value of 0 on the x-axis denotes the moment a country eclipses the CDC threshold of 10 new cases per 100,000 population. This standardization allows for a visual comparison of outbreaks that have occurred on different calendar dates within a country.

The solid lines refer to the speed, or rate of new cases, of the current Omicron-driven outbreak. In each of Botswana, Eswatini, Gabon, Lesotho, Namibia, South Africa, Zambia, and Zimbabwe, the speed of the pandemic has accelerated faster under Omicron than prior VOCs, as depicted by dashed lines.

**Figure 3:**
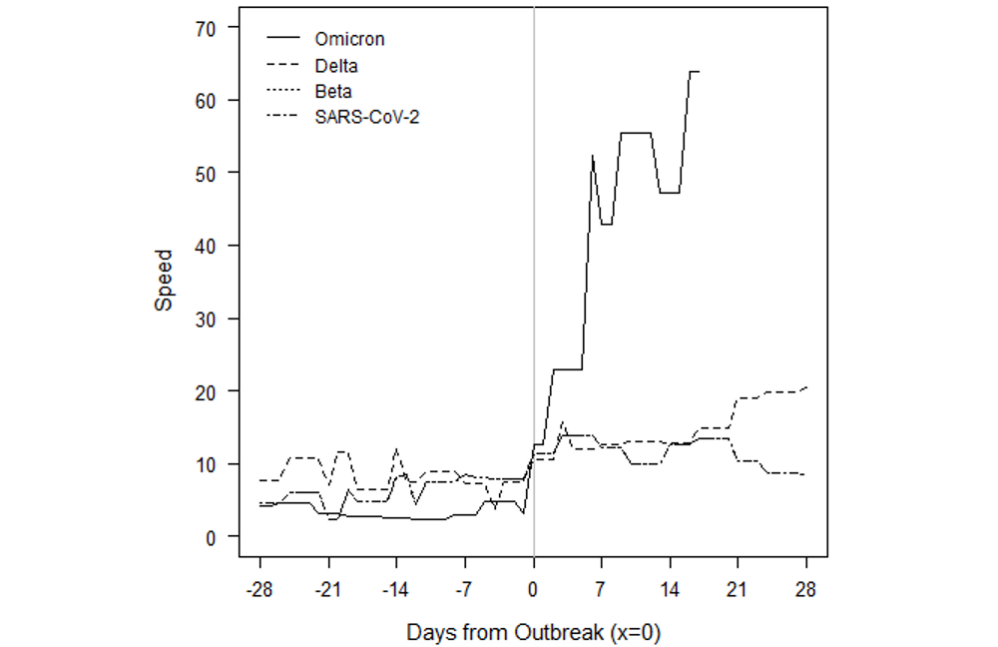
Botswana outbreaks by variant of concern.

In Botswana (Figure 3), the outbreaks from SARS-CoV-2, Beta, and Delta roughly followed the same initial pattern in new cases. Surprisingly, in fact, the progression of SARS-CoV-2 and Beta were similar enough to be almost indistinguishable at the scale of the plot. Data for the country are not always available on a daily basis, so the trend lines contain steps. The Omicron outbreak began with fewer transmissions than Delta, Beta, and the original SARS-CoV-2 variants. Still, before Omicron, every outbreak built slowly over the course of several weeks before peaking. Though not depicted over the time scale, the eventual peaks of Delta, Beta, and SARS-CoV-2 were roughly 96, 20, and 12. The return to sub-outbreak speed was variable because the Beta outbreak had yet to truly subside before the Delta outbreak started. Speed just barely fell to below the CDC threshold before the Delta outbreak.

The Omicron outbreak, in contrast, occurred within less than a week. Speed quickly jumped from roughly 5 to 25 daily new transmissions per 100,000 population. Within 2 weeks, the speed had jumped to 60. If SSA countries with earlier Omicron outbreaks that have subsided are an indication, Botswana may be near its peak speed. The comparison of peak speeds between Omicron and Delta outbreaks is confounded, however, because the Beta outbreak had yet to truly subside before the Delta outbreak began.

**Figure 4:**
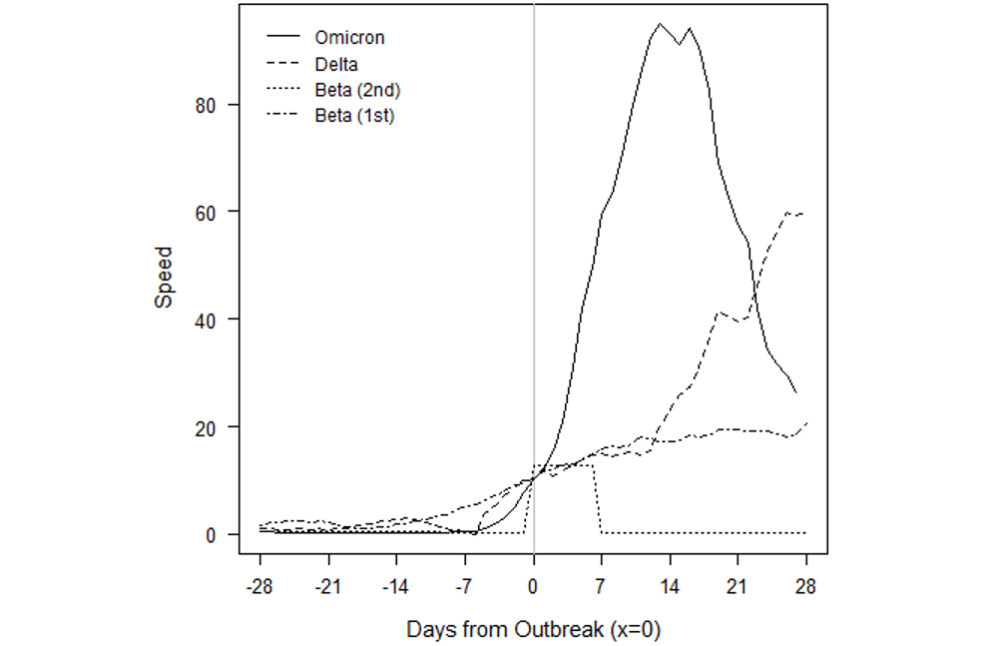
Eswatini outbreaks by variant of concern.

The Omicron outbreak in Eswatini also quickly accelerated from near zero new cases per 100,000 population to a peak of 95 within approximately 2 weeks (Figure 4). The peak is over 1.5 times higher than the previous peak in the Delta outbreak, and 4 times higher than the peak in either of the previous Beta outbreaks. We note that because sequencing data are limited, the assignment of the initial 2 outbreaks to the Beta variant is our own assumption.

**Figure 5:**
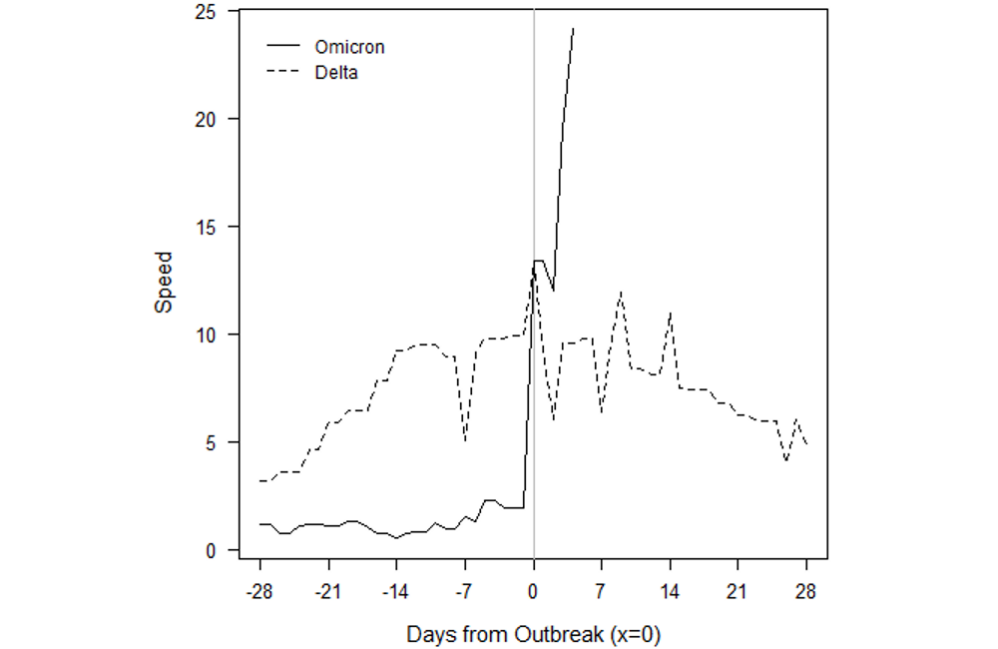
Gabon outbreaks by variant of concern.

Gabon has seen an even higher acceleration of new cases in the Omicron outbreak (Figure 5). From a speed of near zero, the country reached a speed of 25 within less than a week. This peak is already twice that of the earlier Delta outbreak, and acceleration may continue if other SSA countries whose Omicron outbreaks have begun to subside provide any indication. In those countries, the rate of new cases accelerated for at least 2 weeks before the Omicron outbreak began to subside.

**Figure 6:**
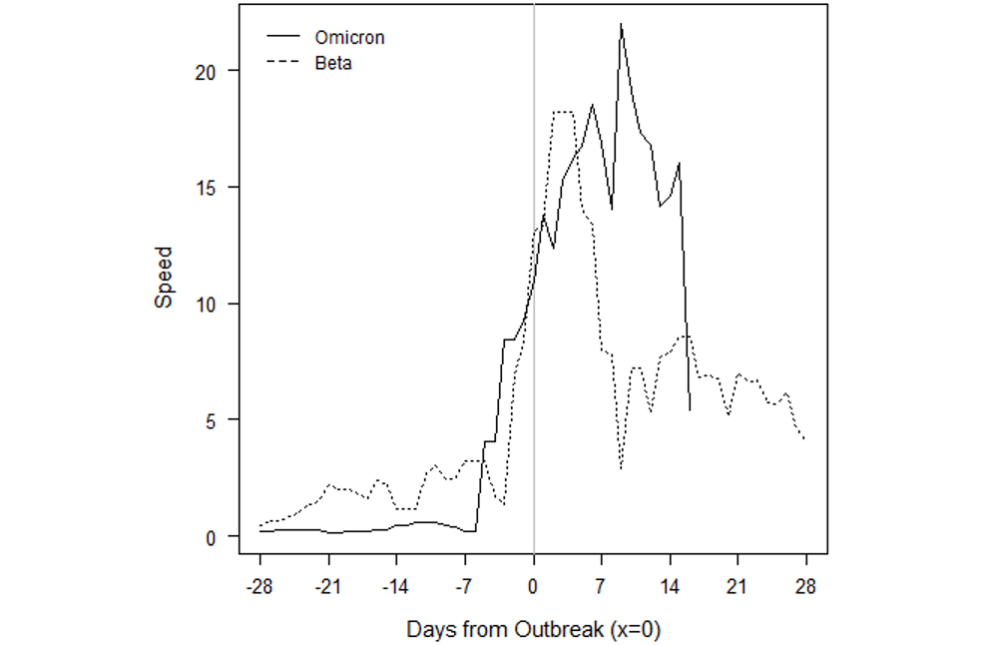
Lesotho outbreaks by variant of concern.

The Omicron and Beta outbreaks in Lesotho followed a broadly similar trajectory, though the duration of the peak speed in Omicron was higher (Figure 6). Each outbreak yielded a peak speed of roughly 20. This similarity of outbreaks by VOC is shared by South Africa. The similarity is perhaps unsurprising because Lesotho has borders contained entirely within South Africa.

**Figure 7:**
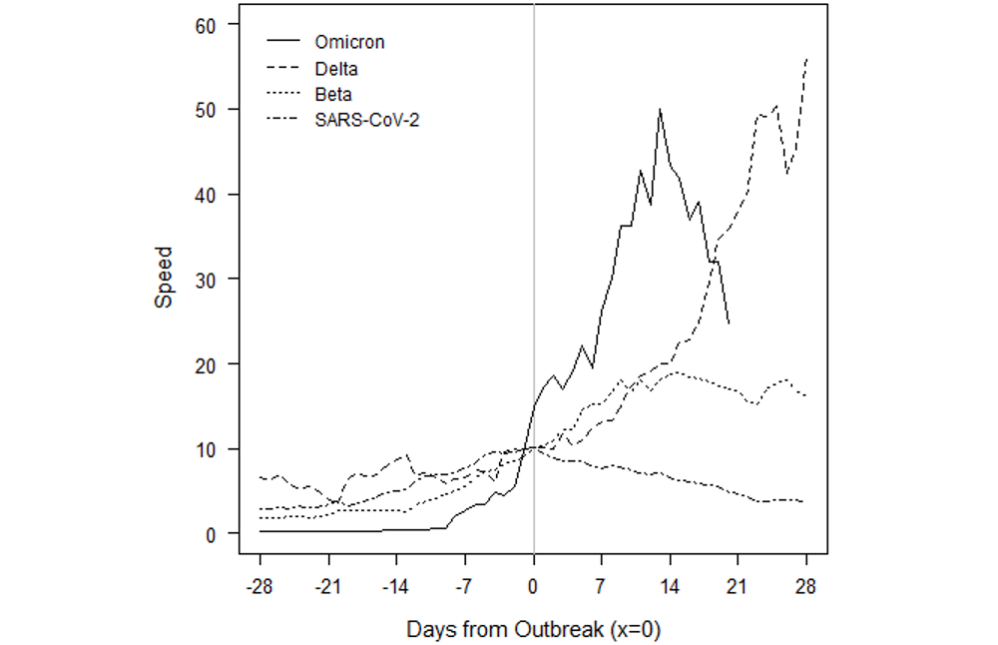
Namibia outbreaks by variant of concern.

Figure 7 depicts the outbreaks for Namibia. Each prior outbreak built slowly over the course of several weeks, while Omicron began with fewer cases than other outbreaks. The initial SARS-CoV-2 outbreak was fleeting, and speed declined immediately after the outbreak threshold was reached. The Beta and Delta outbreaks continued to show positive acceleration for roughly 2 weeks after the CDC threshold was reached. We note that while we attribute the January 2021 outbreak to the Beta variant, which was first detected in the country on January 15, 2021, this attribution is an assumption because sequencing data are limited. Omicron provided slightly more warning than it did for Botswana, but speed jumped dramatically over the course of a week, from roughly 5 to 20, then to 50 within another 10 days. The Omicron outbreak has since subsided as quickly as it rose. The peak speed of 50 is lower than the peak speed of 70 the country eventually reached under the Delta outbreak. However, like in Botswana, the Delta outbreak came at the tail of the Beta outbreak. Speed never fell below 5 between the two, which may confound a comparison of the Omicron and Delta outbreaks.

**Figure 8:**
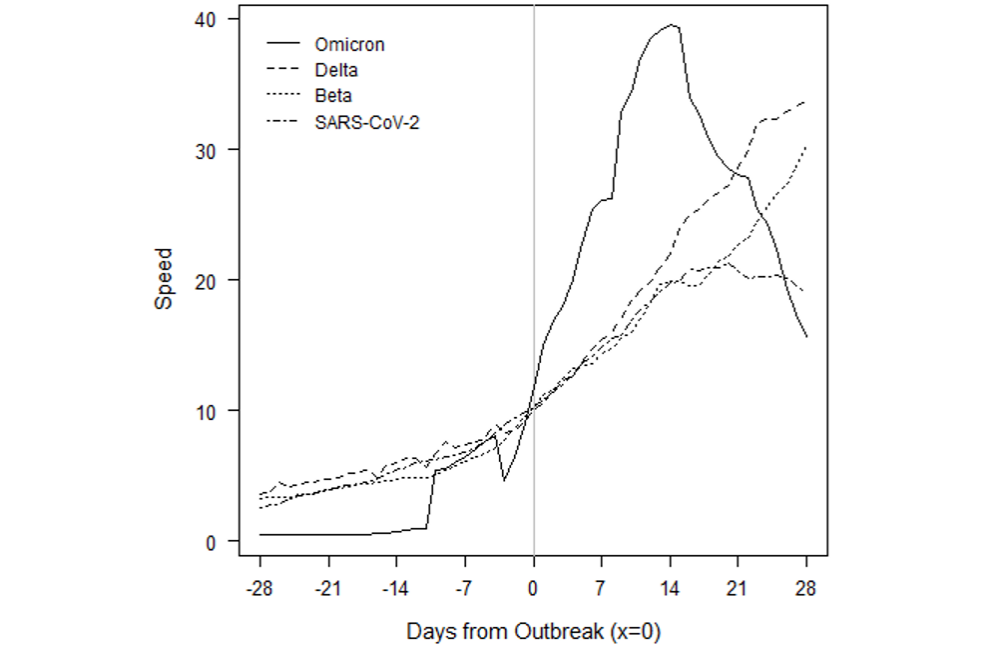
South Africa outbreaks by variant of concern.

South Africa saw a more rapid acceleration in new infections in the Omicron outbreak relative to prior VOCs (Figure 8). The prior outbreaks followed similar patterns. They built slowly over the course of several weeks, but they continued to rise for 2-5 weeks after the CDC outbreak threshold was reached. The Omicron outbreak gave roughly a week and a half of warning. Acceleration has been much faster, and peak speed was higher: 40 in Omicron versus 33, 31, and 20 in Delta, Beta, and SARS-CoV-2, respectively. Speed fell to below 20 by the end of December 2021 and dipped below the outbreak threshold by 0.09 cases per 100,000 population by the second week of January 2022.

**Figure 9:**
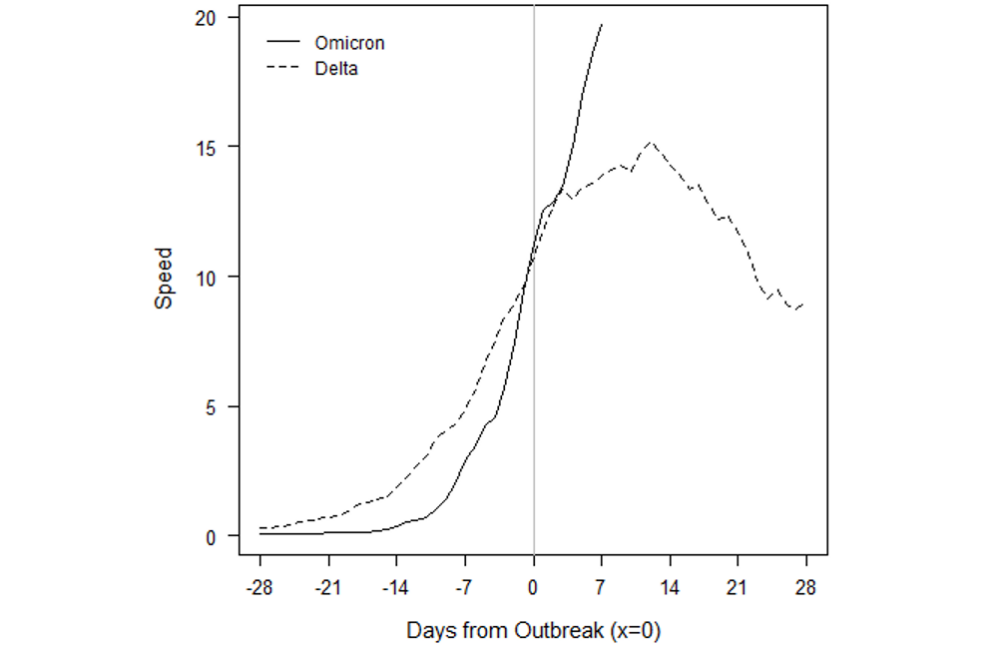
Zambia outbreaks by variant of concern.

Figure 9 depicts the outbreaks in Zambia. The earlier Delta outbreak built slowly over 4 weeks before the country eclipsed the outbreak threshold. Speed slowly rose to 15 over the next 2 weeks before subsiding at roughly the same rate at which it grew. The Omicron outbreak only gave 2 weeks of warning, and within 1 week of surpassing the outbreak threshold, speed had already reached 20.

**Figure 10:**
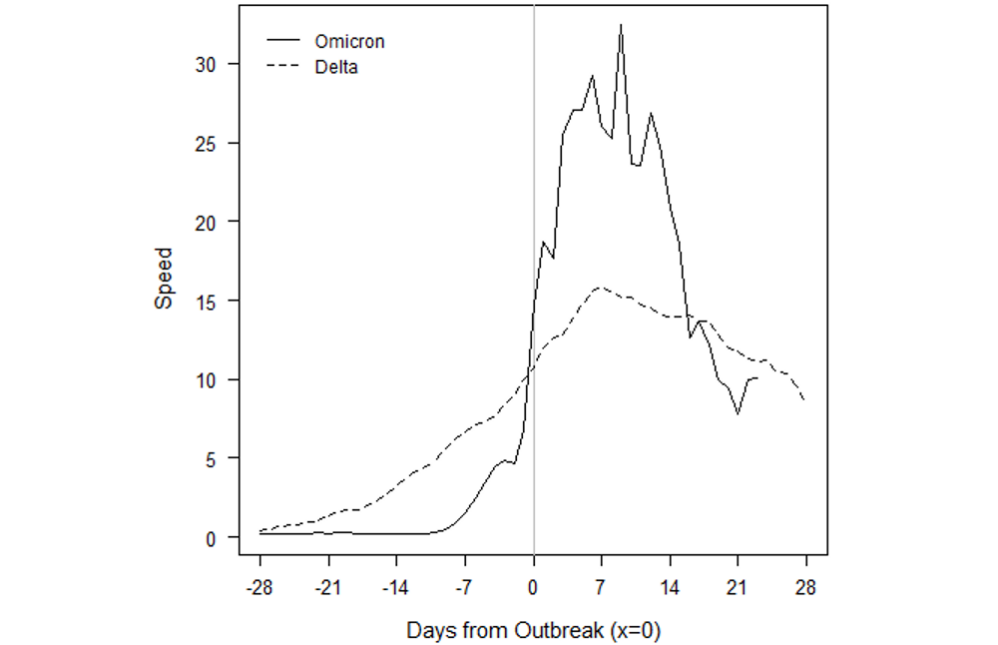
Zimbabwe outbreaks by variant of concern.

Zimbabwe only had one prior outbreak with which to compare Omicron, which is notable because the country started with a lower rate of transmissions in the Omicron outbreak than it did in the Delta and Beta surges in transmissions (Figure 10). Although Beta caused an increase in cases around the start of 2021, speed did not rise enough to reach the CDC outbreak threshold. Delta reached the outbreak threshold after a 3- to 4-week rise in transmissions. Omicron gave only a week’s warning before reaching the threshold and reached a speed over twice the peak of Delta. Again, speed has fallen at roughly the rate at which it grew, but speed reversed its downward trend over the most recent 2 days of data.

Importantly, the raw numbers in Figures 3-10 do not control for cumulative vaccinations or infections. The rate of acceleration under Omicron is even more alarming given the difference in cumulative vaccinations and infections under the Omicron outbreaks and those driven by earlier VOCs. For example, South Africa had reached nearly 12 million total vaccinations by the threshold of the Omicron outbreak, but the country had only surpassed 1.5 million vaccinations by the threshold of the earlier Delta outbreak. The respective numbers for total infections at the start of each outbreak were roughly 3 million and 1.7 million.

Regression analysis provides estimates of the relationship between two variables after controlling for others. We completed Arellano-Bond dynamic panel estimates for SSA over the full calendar year 2021. A dynamic model is required because new SARS-CoV-2 infections are certainly a function of prior infections. The estimation also controls for time-invariant, unobservable characteristics, which may differ by country. We extended the sample period used for surveillance metrics to cover the start of 2021. The extension was needed to examine how Omicron outbreaks compare to those from other VOCs earlier in the year. Note that for countries with complete data for the year, the time period T is slightly higher than 365 days because the model incorporates lags of variables.

The dependent variable is the rate of new cases per 100,000 population. The key independent variable, *after_nov_21*, is an indicator for whether the calendar date is on or beyond November 1, 2021. Because the exact date of origin for Omicron is unknown, this date is meant to be a conservative estimate of the time window in which it originated. However, results are robust to recoding the variable with nearby dates.

The first and seventh lag of the rate of new cases (*lag.pos.1* and *lag.pos.7*, respectively) provide measures of persistence in the pandemic. Their coefficients describe how new cases echo forward from day to day and week to week, respectively. The regression estimates also control for cumulative cases (*cum_cases*), cumulative vaccinations (*cum_vacc*), and an indicator variable for weekend dates (*weekend*), as data reports are less consistently released on weekends.

Because *after_nov_21* is interacted with the lagged rate of new cases, the coefficient on *after_nov_21* by itself does not provide a useful interpretation. To be precise, the coefficient provides the estimated change in the average rate of new cases after November 2021 for a country with 0 previous cases.

However, the interaction between *after_nov_21* and the 1- and 7-day lags of cases (*lag.pos.1*, *lag.pos.7*) confirms that Omicron has changed the evolution of the pandemic. The positive, statistically significant coefficients on the interaction terms mean Omicron has strengthened persistence in the pandemic compared to earlier VOCs. After controlling for vaccinations and prior infections, 1- and 7-day persistence have increased by 1 and 0.52, respectively, since the start of November 1, 2021.

We also note that several SSA countries stand on the precipice of outbreak. The ability to identify a statistical impact of Omicron this early in its spread suggests its true effect may be stronger than the initial results indicate.

The coefficient on *cum_vacc* shows the protective effect of vaccinations at the population level. Over the course of 2021, every vaccination reduces the expected transmission rate by 0.002. The effect is statistically significant at the .05 level. Although its magnitude appears modest, keep in mind the dependent variable measures the *daily* rate of new infections per 100,000.

The positive coefficient on *cum_cases* may come as a surprise if prior infections reduce susceptibility. However, the mechanical correlation between cumulative cases and new infections in an outbreak seems to mask any possible reductions in susceptibility.

Finally, the Arellano-Bond estimator relies on instrumental variables. Although their validity cannot be directly tested, the Sargan test of overidentification restrictions yields a *P* value near 1, which suggests the instruments are valid (Table 2).

**Table 2:** Arellano-Bond dynamic panel data estimates^a^.

Variable	Coefficient (SE)	*P* value
*after_nov_21*	–2.68 (1.02)	.009
*lag.pos.1*	–0.68 (0.14)	<.001
*lag.pos.7*	0.31 (0.31)	.31
*weekend*	–1.12 (0.45)	.01
*cum_cases*	0.008 (0.001)	<.001
*cum_vacc*	–0.002 (0.001)	.03
*after_nov_21* × *lag.pos.1*	0.52 (0.16)	.001
*after_nov_21* × *lag.pos.7*	1.29 (0.28)	<.001

^a^Unbalanced panel: n=43, *t*=359-366, N=15,731. Sargan test: *Χ*^2^_4656_=44 (*P*>.99). Autocorrelation test (1): normal=–1.28 (*P*=.20). Autocorrelation test (2): normal=–0.89 (*P*=.37).

## Discussion

### Principal Findings

Omicron has spread much faster than any previous VOC in SSA. Given the speed of spread, the variant provides relatively little warning of an outbreak based on current surveillance infrastructure and metrics. Although previous VOCs typically provided at least 14-21 days of warning and an average of 35 days, Omicron has driven outbreaks in SSA from a speed of almost zero new cases to 10 per 100,000 population within 7-10 days. The longest warning period of transmission escalation thus far appears to be roughly 10 days, as seen in South Africa, Zambia, and Zimbabwe.

Additionally, Omicron outbreaks have a greater magnitude in terms of the number of new SARS-CoV-2 infections. For the first group of southern African countries to go into outbreak, the peak rate of new transmissions averaged roughly 1.5-2 times the peak of earlier VOCs (Figures 3-10). Botswana and Namibia saw higher peaks under Delta than Omicron, but those comparisons may be confounded because the Delta outbreaks occurred on the tail end of earlier Beta outbreaks.

At the population level, the observed ability for Omicron to outperform prior VOCs is worrisome because total vaccinations and prior infections are higher now than they were when the other VOCs caused outbreaks. Even in countries outside of SSA with high vaccination rates, there have been sharp increases in the spread of SARS-CoV-2 infections caused by Omicron [45]. In the United Kingdom, Omicron has been shown to be associated with a 5.41-fold higher risk of reinfection compared to Delta, confirming relatively low levels of immunity from prior infections [46]. Similar reinfection rates have been reported in South Africa [47]. Both vaccinations and prior infections are expected to reduce susceptibility in the population. However, our findings suggest that the degree of protection may be lessened against Omicron (Table 2).

The results therefore highlight the enhanced transmissibility of Omicron compared to other VOCs. Still, the results do not precisely identify the extent to which Omicron may evade vaccines or immunity from prior infections. The data are too early to inform whether Omicron outbreaks are driven by new infections or reinfections in either the vaccinated or unvaccinated population [47].

Likewise, the coming weeks will provide critical information on the effect of Omicron outbreaks on deaths and hospitalizations. Recent data from the United Kingdom indicate SARS-CoV-2 infections from the Omicron variant may not be any less severe than the Delta variant [46], which contradicts observations from South Africa that suggest Omicron infections are less severe than earlier VOC infections [48,49]. Early laboratory data also show significant reductions in the ability of SARS-CoV-2 antibodies to neutralize the Omicron variant compared to the original virus, among those vaccinated with 2 doses of the Pfizer/BioNTech vaccine [47]. In one study in California, investigators found Omicron increased the risk of hospitalization 4- to 5-fold and increased the risk of symptomatic disease 7- to 10-fold for mRNA vaccine recipients, with similar relative effects for recently vaccinated individuals or individuals with waned antibody titers [49]. Thus, the significantly higher speed and magnitude of an Omicron outbreak, coupled with more extensive vaccine escape [47] and possibly comparable pathogenicity, could signal a potential to significantly overwhelm hospital capacity with a larger number of infected persons within a shorter window of time [45].

In addition to southern Africa being the region where Omicron was first identified, it offers an ideal natural experiment for a comparison of isolated outbreaks driven by Omicron versus prior VOCs. The impact of Omicron may be different for countries already in the midst of current outbreaks driven by another VOC. For example, the United States and the United Kingdom are currently experiencing upsurges in Omicron cases amid ongoing Delta outbreaks. Future work will examine the interaction of VOCs within an outbreak and Omicron’s potential for a viral sweep.

### Limitations

Our study reports on data current up through January 17, 2022. Adverse outcomes are likely to increase because the Omicron outbreak has not had sufficient time to realize morbidity, mortality, severity, transmissibility, and evasiveness. Our data are limited by the granularity of country reporting. Data are reported on a national level for countries within SSA, which precludes intranational analyses that would more closely reflect local regulations and better contextualize national trends. In addition, suboptimal public health infrastructure prevents data from being reported instantaneously and limits the completeness of data. Multiple days of data are frequently bundled into a single report, which may give the impression of zero infections or deaths over a period of days followed by a sudden spike in those same measures. Our data address this issue by calculating 7-day averages per 100,000 population. However, inconsistent reporting in combination with large new daily cases reports can also artificially suppress the 7-day average and mask the true impact of increasing cases over that period.

### Comparison With Prior Work

We conducted prior surveillance estimates and SARS-CoV-2 research on speed, acceleration, jerk, and persistence, relying on dynamic panel data in SSA and other global regions [20,21,23,24,26-29,34,38,50-52].

### Conclusions

Without question, Omicron is more transmissible than prior VOCs [49]. The analysis of outbreaks by VOC in southern African countries shows Omicron transmits at least 2-3 times faster at a country level than prior VOCs. Despite starting from a lower daily rate of SARS-CoV-2 transmissions, Omicron results in worse outbreaks in terms of magnitude by a factor of 1.5-2 on average. However, as Eswatini, Lesotho, Namibia, South Africa, and Zimbabwe have shown, the outbreaks may also subside as quickly as they grow. Still, as outbreaks grow, local surveillance infrastructure may not be able to keep up with the greater number of persons seeking testing for symptoms or exposure. Extensive media coverage of the Omicron variant may also be promoting behavioral changes that could slow the outbreak. Conversely, populations experiencing pandemic fatigue could disregard reports of new outbreaks, relaxing preventative behaviors and leading to additional transmissions. Finally, the recently concluded holiday season likely increased human interactions as it did a year ago. The results presented in this study suggest it takes fewer cases of Omicron to initiate an outbreak than Delta, Beta, Alpha, and the original SARS-CoV-2. Even if Omicron results in lesser disease severity than prior VOCs, hospitals may expect a high caseload of patients because Omicron is highly transmissible. The world should plan how to flatten the curve given the speed, acceleration, jerk, and persistence of Omicron.

## Appendix

Multimedia Appendix 1 Daily SARS-CoV-2 surveillance complete file.

## Abbreviations

CDC: Centers for Disease Control and Prevention
GISAID: Global Initiative on Sharing Avian Influenza Data
SSA: sub-Saharan Africa
VOC: variant of concern
VOI: variant of interest
