# Supplementary material for: Has Omicron Changed the Evolution of the Pandemic?
Source: JMIR Public Health Surveill. 2022 Jan 31;8(1):e35763. doi: 10.2196/35763 (PMC8812144; doi:10.2196/35763)
Supplement: Multimedia Appendix 1 [file publichealth_v8i1e35763_app1.docx]

Multimedia Appendix 1. Daily SARS-CoV-2 surveillance complete file.

| **Country** | **Date** | **New Cases 7-Day Moving Average** | **New Cases/100K 7-Day Moving Average** | **Total Cases** | **Total Cases/ 100K** | **New Deaths 7-Day Moving Average** | **New Deaths per 100K 7-Day Moving Average** | **Total Deaths** | **Total Deaths/ 100K** | **7-Day Persistence (Fridays only)** | **Acceleration Weekly** | **Jerk Weekly** | **Exponential Growth Potential Weekly** |
| --- | --- | --- | --- | --- | --- | --- | --- | --- | --- | --- | --- | --- | --- |
| Angola | Fri, December 03, 2021 | 13 | 0 | 65,223 | 192 | 0 | 0 | 1,735 | 5 | 0 | -0 | -0 | -0 |
| Angola | Fri, December 10, 2021 | 21 | 0 | 65,371 | 193 | 0 | 0 | 1,737 | 5 | 0 | 0 | 0 | 0 |
| Angola | Fri, December 17, 2021 | 56 | 0 | 65,760 | 194 | 0 | 0 | 1,738 | 5 | 0 | 1 | 1 | 1 |
| Angola | Fri, December 24, 2021 | 637 | 2 | 70,221 | 207 | 1 | 0 | 1,746 | 5 | 0 | 12 | 11 | 13 |
| Angola | Fri, December 31, 2021 | 1,625 | 5 | 81,593 | 240 | 3 | 0 | 1,770 | 5 | 3 | 20 | 8 | 26 |
| Angola | Fri, January 07, 2022 | 1,026 | 3 | 88,775 | 262 | 6 | 0 | 1,809 | 5 | 7 | -12 | -33 | -16 |
| Angola | Fri, January 14, 2022 | 647 | 2 | 93,302 | 275 | 6 | 0 | 1,852 | 5 | 2 | -8 | 5 | -10 |
| Botswana | Fri, December 03, 2021 | 56 | 2 | 195,302 | 8,147 | 0 | 0 | 2,419 | 101 | 1 | -3 | 9 | -7 |
| Botswana | Fri, December 10, 2021 | 113 | 5 | 196,090 | 8,180 | 0 | 0 | 2,421 | 101 | 4 | 16 | 19 | 23 |
| Botswana | Fri, December 17, 2021 | 539 | 22 | 199,864 | 8,337 | 1 | 0 | 2,425 | 101 | 6 | 125 | 108 | 140 |
| Botswana | Fri, December 24, 2021 | 1,304 | 54 | 208,994 | 8,718 | 0 | 0 | 2,427 | 101 | 30 | 223 | 99 | 292 |
| Botswana | Fri, December 31, 2021 | 1,502 | 63 | 219,509 | 9,157 | 2 | 0 | 2,444 | 102 | 73 | 58 | -166 | 159 |
| Botswana | Fri, January 07, 2022 | 1,478 | 62 | 229,855 | 9,588 | 4 | 0 | 2,475 | 103 | 85 | -7 | -65 | -55 |
| Botswana | Fri, January 14, 2022 | 1,118 | 47 | 237,678 | 9,915 | 6 | 0 | 2,514 | 105 | 30 | -105 | -98 | -185 |
| Burkina Faso | Fri, December 03, 2021 | 69 | 0 | 16,000 | 74 | 3 | 0 | 286 | 1 | 0 | 2 | 3 | 2 |
| Burkina Faso | Fri, December 10, 2021 | 48 | 0 | 16,334 | 76 | 1 | 0 | 290 | 1 | 1 | -1 | -3 | -1 |
| Burkina Faso | Fri, December 17, 2021 | 48 | 0 | 16,672 | 78 | 1 | 0 | 296 | 1 | 0 | 0 | 1 | 0 |
| Burkina Faso | Fri, December 24, 2021 | 53 | 0 | 17,040 | 79 | 3 | 0 | 317 | 1 | 0 | 0 | 0 | 0 |
| Burkina Faso | Fri, December 31, 2021 | 85 | 0 | 17,632 | 82 | 0 | 0 | 318 | 1 | 0 | 1 | 1 | 2 |
| Burkina Faso | Fri, January 07, 2022 | 178 | 1 | 18,878 | 88 | 2 | 0 | 333 | 2 | 1 | 3 | 2 | 4 |
| Burkina Faso | Fri, January 14, 2022 | 156 | 1 | 19,972 | 93 | 1 | 0 | 339 | 2 | 0 | -1 | -4 | -2 |
| Burundi | Fri, December 03, 2021 | 8 | 0 | 20,439 | 167 | 0 | 0 | 38 | 0 | 0 | -0 | 0 | -0 |
| Burundi | Fri, December 10, 2021 | 32 | 0 | 20,666 | 169 | 0 | 0 | 38 | 0 | 0 | 1 | 1 | 2 |
| Burundi | Fri, December 17, 2021 | 108 | 1 | 21,422 | 175 | 0 | 0 | 38 | 0 | 0 | 4 | 3 | 5 |
| Burundi | Fri, December 24, 2021 | 319 | 3 | 23,657 | 193 | 0 | 0 | 38 | 0 | 1 | 12 | 8 | 15 |
| Burundi | Fri, December 31, 2021 | 530 | 4 | 27,366 | 223 | 0 | 0 | 38 | 0 | 4 | 12 | -0 | 19 |
| Burundi | Fri, January 07, 2022 | 1,051 | 9 | 34,722 | 283 | 0 | 0 | 38 | 0 | 6 | 30 | 18 | 42 |
| Burundi | Fri, January 14, 2022 | 219 | 2 | 36,257 | 296 | 0 | 0 | 38 | 0 | 4 | -47 | -77 | -24 |
| Cabo Verde | Fri, December 03, 2021 | 10 | 2 | 38,426 | 6,839 | 0 | 0 | 351 | 62 | 0 | 7 | 7 | 10 |
| Cabo Verde | Fri, December 10, 2021 | 9 | 2 | 38,489 | 6,850 | 0 | 0 | 351 | 62 | 3 | -2 | -9 | -4 |
| Cabo Verde | Fri, December 17, 2021 | 10 | 2 | 38,557 | 6,862 | 0 | 0 | 351 | 62 | 2 | 1 | 3 | 3 |
| Cabo Verde | Fri, December 24, 2021 | 44 | 8 | 38,865 | 6,917 | 0 | 0 | 352 | 63 | 2 | 43 | 42 | 48 |
| Cabo Verde | Fri, December 31, 2021 | 410 | 73 | 41,732 | 7,427 | 0 | 0 | 352 | 63 | 10 | 455 | 413 | 482 |
| Cabo Verde | Fri, January 07, 2022 | 1,016 | 181 | 48,845 | 8,693 | 1 | 0 | 360 | 64 | 98 | 756 | 300 | 978 |
| Cabo Verde | Fri, January 14, 2022 | 706 | 126 | 53,784 | 9,572 | 1 | 0 | 370 | 66 | 88 | -387 | ##### | -583 |
| Cameroon | Fri, December 03, 2021 | 51 | 0 | 107,148 | 394 | 2 | 0 | 1,804 | 7 | 0 | -1 | -3 | -1 |
| Cameroon | Fri, December 10, 2021 | 57 | 0 | 107,549 | 395 | 3 | 0 | 1,823 | 7 | 0 | 0 | 1 | 1 |
| Cameroon | Fri, December 17, 2021 | 16 | 0 | 107,662 | 395 | 2 | 0 | 1,836 | 7 | 0 | -1 | -1 | -1 |
| Cameroon | Fri, December 24, 2021 | 113 | 0 | 108,451 | 398 | 2 | 0 | 1,851 | 7 | 0 | 2 | 4 | 3 |
| Cameroon | Fri, December 31, 2021 | 131 | 0 | 109,367 | 402 | 0 | 0 | 1,851 | 7 | 1 | 0 | -2 | 1 |
| Cameroon | Fri, January 07, 2022 | 43 | 0 | 109,666 | 403 | 0 | 0 | 1,853 | 7 | 1 | -2 | -3 | -2 |
| Cameroon | Fri, January 14, 2022 | 0 | 0 | 109,666 | 403 | 0 | 0 | 1,853 | 7 | 0 | -1 | 1 | 0 |
| Central African Republic | Fri, December 03, 2021 | 5 | 0 | 11,742 | 239 | 0 | 0 | 101 | 2 | 0 | -0 | -1 | -0 |
| Central African Republic | Fri, December 10, 2021 | 24 | 0 | 11,912 | 242 | 0 | 0 | 101 | 2 | 0 | 3 | 3 | 3 |
| Central African Republic | Fri, December 17, 2021 | 7 | 0 | 11,961 | 243 | 0 | 0 | 101 | 2 | 1 | -2 | -5 | -2 |
| Central African Republic | Fri, December 24, 2021 | 0 | 0 | 11,961 | 243 | 0 | 0 | 101 | 2 | 0 | -1 | 1 | 0 |
| Central African Republic | Fri, December 31, 2021 | 29 | 1 | 12,163 | 247 | 0 | 0 | 101 | 2 | 0 | 4 | 5 | 4 |
| Central African Republic | Fri, January 07, 2022 | 42 | 1 | 12,454 | 253 | 0 | 0 | 101 | 2 | 1 | 2 | -2 | 3 |
| Central African Republic | Fri, January 14, 2022 | 124 | 3 | 13,319 | 271 | 1 | 0 | 108 | 2 | 0 | 12 | 10 | 14 |
| Chad | Fri, December 03, 2021 | 85 | 1 | 5,703 | 34 | 1 | 0 | 181 | 1 | 0 | 4 | 4 | 4 |
| Chad | Fri, December 10, 2021 | 0 | 0 | 5,703 | 34 | 0 | 0 | 181 | 1 | 1 | -4 | -7 | 0 |
| Chad | Fri, December 17, 2021 | 0 | 0 | 5,703 | 34 | 0 | 0 | 181 | 1 | 0 | 0 | 4 | 0 |
| Chad | Fri, December 24, 2021 | 0 | 0 | 5,703 | 34 | 0 | 0 | 181 | 1 | 0 | 0 | 0 | 0 |
| Chad | Fri, December 31, 2021 | 0 | 0 | 5,703 | 34 | 0 | 0 | 181 | 1 | 0 | 0 | 0 | 0 |
| Chad | Fri, January 07, 2022 | 69 | 0 | 6,185 | 37 | 0 | 0 | 184 | 1 | 0 | 3 | 3 | 3 |
| Chad | Fri, January 14, 2022 | 53 | 0 | 6,558 | 39 | 0 | 0 | 185 | 1 | 0 | -1 | -3 | -1 |
| Comoros | Fri, December 03, 2021 | 6 | 1 | 4,527 | 510 | 0 | 0 | 150 | 17 |  | 0 | 3 | 0 |
| Comoros | Fri, December 10, 2021 | 5 | 1 | 4,560 | 513 | 0 | 0 | 151 | 17 |  | -1 | -1 | -2 |
| Comoros | Fri, December 17, 2021 | 7 | 1 | 4,606 | 518 | 0 | 0 | 151 | 17 |  | 1 | 3 | 3 |
| Comoros | Fri, December 24, 2021 | 104 | 12 | 5,336 | 601 | 0 | 0 | 153 | 17 |  | 77 | 76 | 80 |
| Comoros | Fri, December 31, 2021 | 168 | 19 | 6,515 | 733 | 1 | 0 | 157 | 18 |  | 51 | -26 | 82 |
| Comoros | Fri, January 07, 2022 | 140 | 16 | 7,497 | 844 | 0 | 0 | 159 | 18 |  | -22 | -73 | -50 |
| Comoros | Fri, January 14, 2022 | 35 | 4 | 7,745 | 872 | 0 | -0 | 159 | 18 |  | -83 | -60 | -48 |
| Côte d’Ivoire | Fri, December 03, 2021 | 15 | 0 | 61,794 | 228 | 0 | 0 | 705 | 3 | 0 | -0 | -0 | -0 |
| Côte d’Ivoire | Fri, December 10, 2021 | 13 | 0 | 61,882 | 229 | 0 | 0 | 706 | 3 | 0 | -0 | -0 | -0 |
| Côte d’Ivoire | Fri, December 17, 2021 | 21 | 0 | 62,032 | 229 | 0 | 0 | 706 | 3 | 0 | 0 | 0 | 0 |
| Côte d’Ivoire | Fri, December 24, 2021 | 194 | 1 | 63,392 | 234 | 0 | 0 | 707 | 3 | 0 | 4 | 4 | 5 |
| Côte d’Ivoire | Fri, December 31, 2021 | 1,087 | 4 | 71,004 | 262 | 1 | 0 | 714 | 3 | 1 | 23 | 19 | 25 |
| Côte d’Ivoire | Fri, January 07, 2022 | 760 | 3 | 76,321 | 282 | 3 | 0 | 732 | 3 | 5 | -8 | -32 | -13 |
| Côte d’Ivoire | Fri, January 14, 2022 | 340 | 1 | 78,699 | 291 | 2 | 0 | 748 | 3 | 1 | -11 | -2 | -10 |
| Democratic Republic of Congo | Fri, December 03, 2021 | 29 | 0 | 58,319 | 63 | 0 | 0 | 1,107 | 1 | 0 | -0 | -0 | -0 |
| Democratic Republic of Congo | Fri, December 10, 2021 | 219 | 0 | 59,851 | 65 | 2 | 0 | 1,118 | 1 | 0 | 1 | 1 | 2 |
| Democratic Republic of Congo | Fri, December 17, 2021 | 657 | 1 | 64,448 | 70 | 1 | 0 | 1,126 | 1 | 0 | 3 | 2 | 4 |
| Democratic Republic of Congo | Fri, December 24, 2021 | 802 | 1 | 70,059 | 76 | 0 | 0 | 1,126 | 1 | 1 | 1 | -2 | 3 |
| Democratic Republic of Congo | Fri, December 31, 2021 | 676 | 1 | 74,793 | 81 | 11 | 0 | 1,205 | 1 | 1 | -1 | -2 | -2 |
| Democratic Republic of Congo | Fri, January 07, 2022 | 769 | 1 | 80,175 | 87 | 3 | 0 | 1,225 | 1 | 1 | 1 | 2 | 2 |
| Democratic Republic of Congo | Fri, January 14, 2022 | 304 | 0 | 82,306 | 89 | 0 | 0 | 1,225 | 1 | 0 | -4 | -4 | -3 |
| Equatorial Guinea | Fri, December 03, 2021 | 3 | 0 | 13,599 | 938 | 0 | 0 | 175 | 12 | 0 | -2 | -3 | -2 |
| Equatorial Guinea | Fri, December 10, 2021 | 2 | 0 | 13,612 | 939 | 0 | 0 | 175 | 12 | 0 | -0 | 2 | -1 |
| Equatorial Guinea | Fri, December 17, 2021 | 1 | 0 | 13,618 | 939 | 0 | 0 | 175 | 12 | 0 | -0 | -0 | -0 |
| Equatorial Guinea | Fri, December 24, 2021 | 1 | 0 | 13,623 | 940 | 0 | 0 | 175 | 12 | 0 | -0 | 0 | -0 |
| Equatorial Guinea | Fri, December 31, 2021 | 12 | 1 | 13,710 | 946 | 0 | 0 | 175 | 12 | 0 | 6 | 6 | 6 |
| Equatorial Guinea | Fri, January 07, 2022 | 99 | 7 | 14,401 | 993 | 0 | 0 | 177 | 12 | 1 | 42 | 36 | 45 |
| Equatorial Guinea | Fri, January 14, 2022 | 131 | 9 | 15,319 | 1,057 | 0 | 0 | 178 | 12 | 3 | 16 | -26 | 31 |
| Eritrea | Fri, December 03, 2021 | 22 | 1 | 7,442 | 207 | 0 | 0 | 61 | 2 |  | 0 | -1 | 1 |
| Eritrea | Fri, December 10, 2021 | 25 | 1 | 7,616 | 211 | 0 | 0 | 63 | 2 |  | 0 | 0 | 2 |
| Eritrea | Fri, December 17, 2021 | 16 | 0 | 7,731 | 215 | 1 | 0 | 67 | 2 |  | -2 | -2 | -2 |
| Eritrea | Fri, December 24, 2021 | 17 | 0 | 7,853 | 218 | 1 | 0 | 72 | 2 |  | 0 | 2 | 1 |
| Eritrea | Fri, December 31, 2021 | 23 | 1 | 8,011 | 222 | 1 | 0 | 76 | 2 |  | 1 | 1 | 2 |
| Eritrea | Fri, January 07, 2022 | 36 | 1 | 8,262 | 229 | 1 | 0 | 80 | 2 |  | 3 | 2 | 4 |
| Eritrea | Fri, January 14, 2022 | 83 | 2 | 8,845 | 246 | 1 | 0 | 85 | 2 |  | 9 | 7 | 12 |
| Ethiopia | Fri, December 03, 2021 | 127 | 0 | 371,946 | 316 | 9 | 0 | 6,787 | 6 | 0 | -0 | 0 | -0 |
| Ethiopia | Fri, December 10, 2021 | 132 | 0 | 372,868 | 316 | 5 | 0 | 6,822 | 6 | 0 | 0 | 0 | 0 |
| Ethiopia | Fri, December 17, 2021 | 307 | 0 | 375,019 | 318 | 5 | 0 | 6,859 | 6 | 0 | 1 | 1 | 1 |
| Ethiopia | Fri, December 24, 2021 | 2,245 | 2 | 390,737 | 331 | 4 | 0 | 6,888 | 6 | 0 | 12 | 10 | 12 |
| Ethiopia | Fri, December 31, 2021 | 4,229 | 4 | 420,342 | 357 | 7 | 0 | 6,937 | 6 | 3 | 12 | 0 | 17 |
| Ethiopia | Fri, January 07, 2022 | 3,121 | 3 | 442,187 | 375 | 11 | 0 | 7,012 | 6 | 5 | -7 | -18 | -11 |
| Ethiopia | Fri, January 14, 2022 | 1,767 | 1 | 454,556 | 386 | 15 | 0 | 7,117 | 6 | 1 | -8 | -1 | -9 |
| Gabon | Fri, December 03, 2021 | 24 | 1 | 37,391 | 1,641 | 0 | 0 | 279 | 12 | 1 | -5 | -2 | -6 |
| Gabon | Fri, December 10, 2021 | 23 | 1 | 37,551 | 1,648 | 0 | 0 | 281 | 12 | 2 | -0 | 5 | -2 |
| Gabon | Fri, December 17, 2021 | 27 | 1 | 37,743 | 1,656 | 1 | 0 | 285 | 13 | 1 | 1 | 2 | 3 |
| Gabon | Fri, December 24, 2021 | 42 | 2 | 38,039 | 1,669 | 0 | 0 | 286 | 13 | 2 | 5 | 3 | 8 |
| Gabon | Fri, December 31, 2021 | 537 | 24 | 41,798 | 1,834 | 0 | 0 | 288 | 13 | 3 | 152 | 147 | 158 |
| Gabon | Fri, January 07, 2022 | 306 | 13 | 43,939 | 1,928 | 1 | 0 | 295 | 13 | 32 | -71 | -223 | -82 |
| Gabon | Fri, January 14, 2022 | 173 | 8 | 45,152 | 1,981 | 0 | 0 | 297 | 13 | 7 | -41 | 30 | -47 |
| Gambia | Fri, December 03, 2021 | 0 | 0 | 9,992 | 402 | 0 | 0 | 342 | 14 | 0 | 0 | 0 | 0 |
| Gambia | Fri, December 10, 2021 | 1 | 0 | 9,998 | 402 | 0 | 0 | 342 | 14 | 0 | 0 | 0 | 0 |
| Gambia | Fri, December 17, 2021 | 8 | 0 | 10,051 | 404 | 0 | 0 | 342 | 14 | 0 | 2 | 2 | 2 |
| Gambia | Fri, December 24, 2021 | 5 | 0 | 10,087 | 406 | 0 | 0 | 342 | 14 | 0 | -1 | -3 | -1 |
| Gambia | Fri, December 31, 2021 | 12 | 0 | 10,170 | 409 | 0 | 0 | 343 | 14 | 0 | 2 | 3 | 3 |
| Gambia | Fri, January 07, 2022 | 89 | 4 | 10,792 | 434 | 0 | 0 | 343 | 14 | 1 | 22 | 20 | 23 |
| Gambia | Fri, January 14, 2022 | 47 | 2 | 11,122 | 447 | 0 | 0 | 344 | 14 | 2 | -12 | -33 | -12 |
| Ghana | Fri, December 03, 2021 | 0 | 0 | 130,920 | 413 | 0 | 0 | 1,209 | 4 | 0 | -1 | -1 | 0 |
| Ghana | Fri, December 10, 2021 | 47 | 0 | 131,246 | 414 | 3 | 0 | 1,228 | 4 | 0 | 1 | 2 | 1 |
| Ghana | Fri, December 17, 2021 | 95 | 0 | 131,911 | 416 | 4 | 0 | 1,255 | 4 | 0 | 1 | 0 | 1 |
| Ghana | Fri, December 24, 2021 | 378 | 1 | 134,555 | 424 | 2 | 0 | 1,269 | 4 | 0 | 6 | 5 | 7 |
| Ghana | Fri, December 31, 2021 | 1,204 | 4 | 142,986 | 451 | 4 | 0 | 1,295 | 4 | 2 | 18 | 12 | 22 |
| Ghana | Fri, January 07, 2022 | 728 | 2 | 148,079 | 467 | 3 | 0 | 1,313 | 4 | 5 | -11 | -29 | -13 |
| Ghana | Fri, January 14, 2022 | 664 | 2 | 152,729 | 481 | 3 | 0 | 1,336 | 4 | 1 | -1 | 9 | -5 |
| Guinea | Fri, December 03, 2021 | 2 | 0 | 30,770 | 228 | 0 | 0 | 387 | 3 | 0 | -0 | -1 | -0 |
| Guinea | Fri, December 10, 2021 | 4 | 0 | 30,798 | 228 | 0 | 0 | 388 | 3 | 0 | 0 | 0 | 0 |
| Guinea | Fri, December 17, 2021 | 2 | 0 | 30,814 | 228 | 0 | 0 | 388 | 3 | 0 | -0 | -0 | -0 |
| Guinea | Fri, December 24, 2021 | 23 | 0 | 30,976 | 229 | 0 | 0 | 389 | 3 | 0 | 1 | 1 | 1 |
| Guinea | Fri, December 31, 2021 | 154 | 1 | 32,051 | 237 | 0 | 0 | 391 | 3 | 0 | 7 | 6 | 7 |
| Guinea | Fri, January 07, 2022 | 298 | 2 | 34,139 | 253 | 1 | 0 | 397 | 3 | 2 | 8 | 1 | 11 |
| Guinea | Fri, January 14, 2022 | 152 | 1 | 35,202 | 261 | 1 | 0 | 406 | 3 | 1 | -8 | -15 | -8 |
| Guinea-Bissau | Fri, December 03, 2021 | 1 | 0 | 6,444 | 320 | 0 | 0 | 149 | 7 | 0 | 0 | 6 | 0 |
| Guinea-Bissau | Fri, December 10, 2021 | 0 | 0 | 6,447 | 320 | 0 | 0 | 149 | 7 | 0 | -0 | -0 | -0 |
| Guinea-Bissau | Fri, December 17, 2021 | 1 | 0 | 6,455 | 320 | 0 | 0 | 149 | 7 | 0 | 0 | 0 | 0 |
| Guinea-Bissau | Fri, December 24, 2021 | 1 | 0 | 6,462 | 321 | 0 | 0 | 149 | 7 | 0 | -0 | -0 | -0 |
| Guinea-Bissau | Fri, December 31, 2021 | 3 | 0 | 6,484 | 322 | 0 | 0 | 149 | 7 | 0 | 1 | 1 | 1 |
| Guinea-Bissau | Fri, January 07, 2022 | 22 | 1 | 6,638 | 329 | 0 | 0 | 149 | 7 | 0 | 7 | 6 | 7 |
| Guinea-Bissau | Fri, January 14, 2022 | 46 | 2 | 6,962 | 345 | 0 | 0 | 150 | 7 | 1 | 8 | 2 | 12 |
| Kenya | Fri, December 03, 2021 | 64 | 0 | 255,355 | 464 | 0 | 0 | 5,335 | 10 | 0 | 0 | 1 | 1 |
| Kenya | Fri, December 10, 2021 | 82 | 0 | 255,932 | 465 | 1 | 0 | 5,342 | 10 | 0 | 0 | -0 | 0 |
| Kenya | Fri, December 17, 2021 | 605 | 1 | 260,166 | 473 | 2 | 0 | 5,353 | 10 | 0 | 7 | 6 | 7 |
| Kenya | Fri, December 24, 2021 | 2,492 | 5 | 277,609 | 505 | 1 | 0 | 5,357 | 10 | 1 | 24 | 17 | 28 |
| Kenya | Fri, December 31, 2021 | 2,488 | 5 | 295,028 | 537 | 3 | 0 | 5,378 | 10 | 6 | -0 | -24 | -1 |
| Kenya | Fri, January 07, 2022 | 2,015 | 4 | 309,130 | 562 | 7 | 0 | 5,425 | 10 | 6 | -6 | -6 | -12 |
| Kenya | Fri, January 14, 2022 | 1,081 | 2 | 316,700 | 576 | 8 | 0 | 5,482 | 10 | 2 | -12 | -6 | -13 |
| Lesotho | Fri, December 03, 2021 | 12 | 1 | 21,838 | 1,011 | 0 | 0 | 663 | 31 | 0 | 3 | 3 | 3 |
| Lesotho | Fri, December 10, 2021 | 87 | 4 | 22,448 | 1,040 | 0 | 0 | 664 | 31 | 1 | 24 | 22 | 26 |
| Lesotho | Fri, December 17, 2021 | 264 | 12 | 24,298 | 1,125 | 0 | 0 | 665 | 31 | 5 | 57 | 33 | 70 |
| Lesotho | Fri, December 24, 2021 | 471 | 22 | 27,595 | 1,278 | 0 | 0 | 665 | 31 | 16 | 67 | 10 | 101 |
| Lesotho | Fri, December 31, 2021 | 116 | 5 | 28,408 | 1,316 | 0 | 0 | 665 | 31 | 29 | -115 | -182 | -66 |
| Lesotho | Fri, January 07, 2022 | 320 | 15 | 30,651 | 1,420 | 2 | 0 | 678 | 31 | 7 | 66 | 181 | 83 |
| Lesotho | Fri, January 14, 2022 | 136 | 6 | 31,604 | 1,464 | 1 | 0 | 687 | 32 | 7 | -60 | -126 | -51 |
| Liberia | Fri, December 03, 2021 | 0 | 0 | 5,824 | 112 | 0 | 0 | 287 | 6 | 0 | 0 | -0 | 0 |
| Liberia | Fri, December 10, 2021 | 2 | 0 | 5,835 | 113 | 0 | 0 | 287 | 6 | 0 | 0 | 0 | 0 |
| Liberia | Fri, December 17, 2021 | 1 | 0 | 5,844 | 113 | 0 | 0 | 287 | 6 | 0 | -0 | -0 | -0 |
| Liberia | Fri, December 24, 2021 | 12 | 0 | 5,929 | 114 | 0 | 0 | 287 | 6 | 0 | 1 | 2 | 2 |
| Liberia | Fri, December 31, 2021 | 50 | 1 | 6,278 | 121 | 0 | 0 | 287 | 6 | 0 | 5 | 4 | 6 |
| Liberia | Fri, January 07, 2022 | 82 | 2 | 6,851 | 132 | 0 | 0 | 287 | 6 | 1 | 4 | -1 | 7 |
| Liberia | Fri, January 14, 2022 | 39 | 1 | 7,121 | 137 | 0 | 0 | 287 | 6 | 1 | -6 | -10 | -6 |
| Madagascar | Fri, December 03, 2021 | 37 | 0 | 44,330 | 156 | 0 | 0 | 967 | 3 | 0 | -0 | -2 | -1 |
| Madagascar | Fri, December 10, 2021 | 67 | 0 | 44,800 | 158 | 1 | 0 | 972 | 3 | 0 | 1 | 1 | 1 |
| Madagascar | Fri, December 17, 2021 | 142 | 0 | 45,794 | 161 | 1 | 0 | 980 | 3 | 0 | 2 | 1 | 3 |
| Madagascar | Fri, December 24, 2021 | 214 | 1 | 47,295 | 166 | 2 | 0 | 996 | 4 | 1 | 2 | -0 | 3 |
| Madagascar | Fri, December 31, 2021 | 426 | 1 | 50,279 | 177 | 4 | 0 | 1,027 | 4 | 1 | 5 | 3 | 7 |
| Madagascar | Fri, January 07, 2022 | 308 | 1 | 52,434 | 184 | 6 | 0 | 1,067 | 4 | 2 | -3 | -8 | -5 |
| Madagascar | Fri, January 14, 2022 | 238 | 1 | 54,101 | 190 | 7 | 0 | 1,117 | 4 | 1 | -2 | 1 | -3 |
| Malawi | Fri, December 03, 2021 | 9 | 0 | 61,949 | 315 | 0 | 0 | 2,307 | 12 | 0 | 0 | 0 | 0 |
| Malawi | Fri, December 10, 2021 | 28 | 0 | 62,147 | 316 | 0 | 0 | 2,307 | 12 | 0 | 1 | 0 | 1 |
| Malawi | Fri, December 17, 2021 | 257 | 1 | 63,944 | 325 | 1 | 0 | 2,311 | 12 | 0 | 8 | 7 | 9 |
| Malawi | Fri, December 24, 2021 | 827 | 4 | 69,735 | 355 | 2 | 0 | 2,322 | 12 | 2 | 20 | 12 | 24 |
| Malawi | Fri, December 31, 2021 | 763 | 4 | 75,075 | 382 | 6 | 0 | 2,364 | 12 | 6 | -2 | -23 | -8 |
| Malawi | Fri, January 07, 2022 | 572 | 3 | 79,082 | 403 | 6 | 0 | 2,407 | 12 | 5 | -7 | -4 | -12 |
| Malawi | Fri, January 14, 2022 | 454 | 2 | 82,262 | 419 | 7 | 0 | 2,453 | 12 | 1 | -4 | 3 | -8 |
| Mali | Fri, December 03, 2021 | 68 | 0 | 17,698 | 85 | 2 | 0 | 613 | 3 | 0 | 1 | -0 | 1 |
| Mali | Fri, December 10, 2021 | 101 | 0 | 18,404 | 88 | 1 | 0 | 623 | 3 | 1 | 1 | 1 | 2 |
| Mali | Fri, December 17, 2021 | 94 | 0 | 19,064 | 91 | 2 | 0 | 637 | 3 | 1 | -0 | -1 | -1 |
| Mali | Fri, December 24, 2021 | 115 | 1 | 19,866 | 95 | 2 | 0 | 652 | 3 | 1 | 1 | 1 | 2 |
| Mali | Fri, December 31, 2021 | 163 | 1 | 21,008 | 101 | 1 | 0 | 660 | 3 | 1 | 2 | 1 | 3 |
| Mali | Fri, January 07, 2022 | 425 | 2 | 23,980 | 115 | 2 | 0 | 671 | 3 | 1 | 9 | 7 | 11 |
| Mali | Fri, January 14, 2022 | 571 | 3 | 27,980 | 134 | 2 | 0 | 683 | 3 | 1 | 5 | -4 | 10 |
| Mauritania | Fri, December 03, 2021 | 62 | 1 | 39,468 | 827 | 1 | 0 | 837 | 18 | 1 | -2 | -2 | -4 |
| Mauritania | Fri, December 10, 2021 | 39 | 1 | 39,742 | 832 | 2 | 0 | 848 | 18 | 2 | -3 | -2 | -4 |
| Mauritania | Fri, December 17, 2021 | 35 | 1 | 39,990 | 837 | 1 | 0 | 852 | 18 | 1 | -1 | 3 | -2 |
| Mauritania | Fri, December 24, 2021 | 57 | 1 | 40,389 | 846 | 1 | 0 | 861 | 18 | 1 | 3 | 4 | 5 |
| Mauritania | Fri, December 31, 2021 | 155 | 3 | 41,473 | 869 | 1 | 0 | 866 | 18 | 2 | 14 | 11 | 18 |
| Mauritania | Fri, January 07, 2022 | 795 | 17 | 47,036 | 985 | 2 | 0 | 881 | 18 | 4 | 94 | 79 | 105 |
| Mauritania | Fri, January 14, 2022 | 944 | 20 | 53,643 | 1,123 | 3 | 0 | 900 | 19 | 8 | 22 | -72 | 55 |
| Mauritius | Fri, December 03, 2021 | 118 | 9 | 21,868 | 1,717 | 0 | 0 | 240 | 19 | 8 | -14 | -11 | -30 |
| Mauritius | Fri, December 10, 2021 | 85 | 7 | 22,461 | 1,764 | 0 | 0 | 240 | 19 | 15 | -18 | -4 | -29 |
| Mauritius | Fri, December 17, 2021 | 58 | 5 | 22,869 | 1,796 | 0 | 0 | 240 | 19 | 8 | -15 | 4 | -22 |
| Mauritius | Fri, December 24, 2021 | 30 | 2 | 23,077 | 1,812 | 0 | 0 | 240 | 19 |  | -16 | -1 | -16 |
| Mauritius | Fri, December 31, 2021 | 59 | 5 | 23,493 | 1,845 | 0 | 0 | 240 | 19 |  | 16 | 32 | 23 |
| Mauritius | Fri, January 07, 2022 | 56 | 4 | 23,886 | 1,876 | 0 | 0 | 240 | 19 |  | -2 | -18 | -7 |
| Mauritius | Fri, January 14, 2022 | 77 | 6 | 24,425 | 1,918 | 0 | 0 | 240 | 19 |  | 11 | 13 | 22 |
| Mozambique | Fri, December 03, 2021 | 35 | 0 | 151,763 | 472 | 0 | 0 | 1,941 | 6 | 0 | 1 | 1 | 1 |
| Mozambique | Fri, December 10, 2021 | 172 | 1 | 152,965 | 476 | 0 | 0 | 1,942 | 6 | 0 | 3 | 2 | 3 |
| Mozambique | Fri, December 17, 2021 | 735 | 2 | 158,111 | 492 | 1 | 0 | 1,948 | 6 | 1 | 12 | 9 | 14 |
| Mozambique | Fri, December 24, 2021 | 1,709 | 5 | 170,077 | 529 | 2 | 0 | 1,965 | 6 | 3 | 21 | 9 | 28 |
| Mozambique | Fri, December 31, 2021 | 2,715 | 8 | 189,080 | 588 | 6 | 0 | 2,006 | 6 | 7 | 22 | 1 | 36 |
| Mozambique | Fri, January 07, 2022 | 2,456 | 8 | 206,272 | 641 | 10 | 0 | 2,075 | 6 | 12 | -6 | -28 | -17 |
| Mozambique | Fri, January 14, 2022 | 1,650 | 5 | 217,821 | 677 | 6 | 0 | 2,117 | 7 | 4 | -18 | -12 | -25 |
| Namibia | Fri, December 03, 2021 | 51 | 2 | 129,508 | 5,005 | 0 | 0 | 3,573 | 138 | 0 | 11 | 9 | 12 |
| Namibia | Fri, December 10, 2021 | 246 | 10 | 131,230 | 5,072 | 0 | 0 | 3,575 | 138 | 3 | 53 | 42 | 59 |
| Namibia | Fri, December 17, 2021 | 495 | 19 | 134,694 | 5,206 | 1 | 0 | 3,580 | 138 | 11 | 67 | 15 | 95 |
| Namibia | Fri, December 24, 2021 | 1,269 | 49 | 143,576 | 5,549 | 3 | 0 | 3,601 | 139 | 25 | 209 | 142 | 268 |
| Namibia | Fri, December 31, 2021 | 628 | 24 | 147,974 | 5,719 | 5 | 0 | 3,633 | 140 | 66 | -173 | -383 | -172 |
| Namibia | Fri, January 07, 2022 | 506 | 20 | 151,517 | 5,856 | 9 | 0 | 3,698 | 143 | 33 | -33 | 140 | -67 |
| Namibia | Fri, January 14, 2022 | 350 | 14 | 153,969 | 5,951 | 15 | 1 | 3,804 | 147 | 10 | -42 | -9 | -63 |
| Niger | Fri, December 03, 2021 | 20 | 0 | 7,061 | 28 | 1 | 0 | 261 | 1 | 0 | 0 | 0 | 0 |
| Niger | Fri, December 10, 2021 | 13 | 0 | 7,149 | 28 | 1 | 0 | 267 | 1 | 0 | -0 | -0 | -0 |
| Niger | Fri, December 17, 2021 | 5 | 0 | 7,185 | 29 | 1 | 0 | 271 | 1 | 0 | -0 | 0 | -0 |
| Niger | Fri, December 24, 2021 | 11 | 0 | 7,263 | 29 | 0 | 0 | 274 | 1 | 0 | 0 | 0 | 0 |
| Niger | Fri, December 31, 2021 | 20 | 0 | 7,405 | 29 | 0 | 0 | 275 | 1 | 0 | 0 | 0 | 0 |
| Niger | Fri, January 07, 2022 | 69 | 0 | 7,891 | 31 | 1 | 0 | 280 | 1 | 0 | 1 | 1 | 2 |
| Niger | Fri, January 14, 2022 | 65 | 0 | 8,347 | 33 | 1 | 0 | 287 | 1 | 0 | -0 | -1 | -0 |
| Nigeria | Fri, December 03, 2021 | 90 | 0 | 214,513 | 101 | 1 | 0 | 2,980 | 1 | 0 | 0 | 0 | 0 |
| Nigeria | Fri, December 10, 2021 | 277 | 0 | 216,451 | 102 | 0 | 0 | 2,981 | 1 | 0 | 1 | 1 | 1 |
| Nigeria | Fri, December 17, 2021 | 886 | 0 | 222,655 | 105 | 0 | 0 | 2,984 | 1 | 0 | 2 | 1 | 2 |
| Nigeria | Fri, December 24, 2021 | 1,722 | 1 | 234,709 | 111 | 1 | 0 | 2,993 | 1 | 1 | 3 | 1 | 4 |
| Nigeria | Fri, December 31, 2021 | 972 | 0 | 241,513 | 114 | 5 | 0 | 3,030 | 1 | 1 | -2 | -5 | -3 |
| Nigeria | Fri, January 07, 2022 | 785 | 0 | 247,009 | 117 | 6 | 0 | 3,070 | 1 | 1 | -1 | 2 | -1 |
| Nigeria | Fri, January 14, 2022 | 429 | 0 | 250,009 | 118 | 3 | 0 | 3,092 | 1 | 0 | -1 | -1 | -1 |
| Republic of the Congo | Fri, December 03, 2021 | 19 | 0 | 18,970 | 335 | 1 | 0 | 354 | 6 | 0 | 0 | 8 | 1 |
| Republic of the Congo | Fri, December 10, 2021 | 14 | 0 | 19,066 | 337 | 1 | 0 | 359 | 6 | 1 | -1 | -1 | -1 |
| Republic of the Congo | Fri, December 17, 2021 | 16 | 0 | 19,179 | 339 | 1 | 0 | 365 | 6 | 0 | 0 | 1 | 1 |
| Republic of the Congo | Fri, December 24, 2021 | 44 | 1 | 19,490 | 345 | 0 | 0 | 367 | 6 | 0 | 4 | 3 | 4 |
| Republic of the Congo | Fri, December 31, 2021 | 86 | 2 | 20,089 | 355 | 0 | 0 | 367 | 6 | 1 | 5 | 2 | 7 |
| Republic of the Congo | Fri, January 07, 2022 | 251 | 4 | 21,844 | 386 | 0 | 0 | 370 | 7 | 2 | 20 | 15 | 25 |
| Republic of the Congo | Fri, January 14, 2022 | 161 | 3 | 22,969 | 406 | 0 | 0 | 371 | 7 | 2 | -11 | -32 | -15 |
| Rwanda | Fri, December 03, 2021 | 11 | 0 | 100,391 | 756 | 0 | 0 | 1,343 | 10 | 0 | -0 | -0 | -0 |
| Rwanda | Fri, December 10, 2021 | 24 | 0 | 100,556 | 757 | 0 | 0 | 1,344 | 10 | 0 | 1 | 1 | 1 |
| Rwanda | Fri, December 17, 2021 | 90 | 1 | 101,183 | 762 | 0 | 0 | 1,344 | 10 | 0 | 3 | 3 | 4 |
| Rwanda | Fri, December 24, 2021 | 374 | 3 | 103,799 | 782 | 0 | 0 | 1,345 | 10 | 1 | 15 | 12 | 17 |
| Rwanda | Fri, December 31, 2021 | 1,141 | 9 | 111,786 | 842 | 1 | 0 | 1,350 | 10 | 4 | 40 | 25 | 49 |
| Rwanda | Fri, January 07, 2022 | 1,091 | 8 | 119,421 | 899 | 2 | 0 | 1,367 | 10 | 12 | -3 | -43 | -12 |
| Rwanda | Fri, January 14, 2022 | 714 | 5 | 124,419 | 937 | 5 | 0 | 1,399 | 11 | 4 | -20 | -17 | -27 |
| São Tomé and Príncipe | Fri, December 03, 2021 | 0 | 0 | 3,732 | 1,671 | 0 | 0 | 56 | 25 | 0 | 0 | 1 | 0 |
| São Tomé and Príncipe | Fri, December 10, 2021 | 0 | 0 | 3,733 | 1,671 | 0 | 0 | 57 | 26 | 0 | 0 | -0 | 0 |
| São Tomé and Príncipe | Fri, December 17, 2021 | 0 | 0 | 3,735 | 1,672 | 0 | 0 | 57 | 26 | 0 | 0 | 0 | 1 |
| São Tomé and Príncipe | Fri, December 24, 2021 | 1 | 1 | 3,744 | 1,676 | 0 | 0 | 57 | 26 | 0 | 3 | 3 | 4 |
| São Tomé and Príncipe | Fri, December 31, 2021 | 22 | 10 | 3,897 | 1,745 | 0 | 0 | 57 | 26 | 1 | 64 | 61 | 66 |
| São Tomé and Príncipe | Fri, January 07, 2022 | 74 | 33 | 4,416 | 1,977 | 1 | 0 | 62 | 28 | 13 | 164 | 99 | 195 |
| São Tomé and Príncipe | Fri, January 14, 2022 | 149 | 67 | 5,458 | 2,444 | 0 | 0 | 65 | 29 | 16 | 234 | 70 | 331 |
| Senegal | Fri, December 03, 2021 | 5 | 0 | 74,007 | 430 | 0 | 0 | 1,886 | 11 | 0 | 0 | 0 | 0 |
| Senegal | Fri, December 10, 2021 | 8 | 0 | 74,065 | 431 | 0 | 0 | 1,886 | 11 | 0 | 0 | 0 | 0 |
| Senegal | Fri, December 17, 2021 | 9 | 0 | 74,129 | 431 | 0 | 0 | 1,886 | 11 | 0 | 0 | -0 | 0 |
| Senegal | Fri, December 24, 2021 | 25 | 0 | 74,302 | 432 | 1 | 0 | 1,890 | 11 | 0 | 1 | 1 | 1 |
| Senegal | Fri, December 31, 2021 | 108 | 1 | 75,055 | 436 | 0 | 0 | 1,890 | 11 | 0 | 3 | 3 | 4 |
| Senegal | Fri, January 07, 2022 | 411 | 2 | 77,935 | 453 | 1 | 0 | 1,895 | 11 | 1 | 12 | 9 | 14 |
| Senegal | Fri, January 14, 2022 | 562 | 3 | 81,868 | 476 | 1 | 0 | 1,904 | 11 | 1 | 6 | -6 | 12 |
| Seychelles | Fri, December 03, 2021 | 57 | 58 | 23,599 | 23,859 | 0 | 0 | 127 | 128 |  | 101 | 61 | 203 |
| Seychelles | Fri, December 10, 2021 | 30 | 30 | 23,806 | 24,068 | 0 | 0 | 129 | 130 |  | -197 | -298 | -203 |
| Seychelles | Fri, December 17, 2021 | 34 | 35 | 24,047 | 24,312 | 0 | 0 | 131 | 132 |  | 34 | 232 | 92 |
| Seychelles | Fri, December 24, 2021 | 29 | 29 | 24,249 | 24,516 | 0 | 0 | 131 | 132 |  | -39 | -74 | -90 |
| Seychelles | Fri, December 31, 2021 | 77 | 78 | 24,788 | 25,061 | 0 | 0 | 134 | 135 |  | 341 | 380 | 431 |
| Seychelles | Fri, January 07, 2022 | 311 | 315 | 26,968 | 27,265 | 0 | 0 | 134 | 135 |  | 1,659 | 1,318 | 1,912 |
| Seychelles | Fri, January 14, 2022 | 590 | 597 | 31,098 | 31,441 | 0 | 0 | 136 | 137 |  | 1,971 | 312 | 2,869 |
| Sierra Leone | Fri, December 03, 2021 | 0 | 0 | 6,402 | 79 | 0 | 0 | 121 | 1 | 0 | 0 | -0 | 0 |
| Sierra Leone | Fri, December 10, 2021 | 3 | 0 | 6,421 | 79 | 0 | 0 | 121 | 1 | 0 | 0 | 0 | 0 |
| Sierra Leone | Fri, December 17, 2021 | 4 | 0 | 6,452 | 79 | 0 | 0 | 121 | 1 | 0 | 0 | -0 | 0 |
| Sierra Leone | Fri, December 24, 2021 | 30 | 0 | 6,659 | 82 | 0 | 0 | 123 | 2 | 0 | 2 | 2 | 2 |
| Sierra Leone | Fri, December 31, 2021 | 58 | 1 | 7,064 | 87 | 0 | 0 | 123 | 2 | 0 | 2 | 0 | 3 |
| Sierra Leone | Fri, January 07, 2022 | 45 | 1 | 7,382 | 91 | 0 | 0 | 123 | 2 | 1 | -1 | -4 | -2 |
| Sierra Leone | Fri, January 14, 2022 | 17 | 0 | 7,500 | 92 | 0 | 0 | 125 | 2 | 0 | -2 | -1 | -2 |
| Somalia | Fri, December 03, 2021 | 5 | 0 | 23,051 | 141 | 1 | 0 | 1,331 | 8 | 0 | -0 | 0 | -0 |
| Somalia | Fri, December 10, 2021 | 3 | 0 | 23,074 | 141 | 0 | 0 | 1,333 | 8 | 0 | -0 | -0 | -0 |
| Somalia | Fri, December 17, 2021 | 14 | 0 | 23,169 | 142 | 0 | 0 | 1,333 | 8 | 0 | 0 | 1 | 1 |
| Somalia | Fri, December 24, 2021 | 52 | 0 | 23,532 | 144 | 0 | 0 | 1,333 | 8 | 0 | 2 | 1 | 2 |
| Somalia | Fri, December 31, 2021 | 0 | 0 | 23,532 | 144 | 0 | 0 | 1,333 | 8 | 0 | -2 | -4 | 0 |
| Somalia | Fri, January 07, 2022 | 104 | 1 | 24,261 | 148 | 0 | 0 | 1,335 | 8 | 0 | 4 | 7 | 4 |
| Somalia | Fri, January 14, 2022 | 0 | 0 | 24,261 | 148 | 0 | 0 | 1,335 | 8 | 0 | -4 | -9 | 0 |
| South Africa | Fri, December 03, 2021 | 6,982 | 12 | ####### | 4,973 | 23 | 0 | 89,944 | 150 | 2 | 67 | 58 | 74 |
| South Africa | Fri, December 10, 2021 | 15,467 | 26 | ####### | 5,153 | 19 | 0 | 90,080 | 150 | 19 | 99 | 32 | 134 |
| South Africa | Fri, December 17, 2021 | 23,437 | 39 | ####### | 5,426 | 31 | 0 | 90,297 | 150 | 30 | 93 | -6 | 159 |
| South Africa | Fri, December 24, 2021 | 16,654 | 28 | ####### | 5,620 | 64 | 0 | 90,743 | 151 | 52 | -79 | -172 | -124 |
| South Africa | Fri, December 31, 2021 | 9,311 | 16 | ####### | 5,729 | 57 | 0 | 91,145 | 152 | 37 | -86 | -7 | -96 |
| South Africa | Fri, January 07, 2022 | 7,932 | 13 | ####### | 5,821 | 79 | 0 | 91,701 | 153 | 21 | -16 | 70 | -39 |
| South Africa | Fri, January 14, 2022 | 5,461 | 9 | ####### | 5,885 | 124 | 0 | 92,566 | 154 | 6 | -29 | -13 | -43 |
| South Sudan | Fri, December 03, 2021 | 12 | 0 | 12,804 | 112 | 0 | 0 | 133 | 1 | 0 | 0 | 0 | 0 |
| South Sudan | Fri, December 10, 2021 | 10 | 0 | 12,873 | 113 | 0 | 0 | 133 | 1 | 0 | -0 | -0 | -0 |
| South Sudan | Fri, December 17, 2021 | 62 | 1 | 13,309 | 117 | 0 | 0 | 133 | 1 | 0 | 3 | 3 | 4 |
| South Sudan | Fri, December 24, 2021 | 181 | 2 | 14,577 | 128 | 0 | 0 | 135 | 1 | 1 | 7 | 4 | 9 |
| South Sudan | Fri, December 31, 2021 | 95 | 1 | 15,242 | 134 | 0 | 0 | 135 | 1 | 2 | -5 | -13 | -6 |
| South Sudan | Fri, January 07, 2022 | 114 | 1 | 16,042 | 141 | 0 | 0 | 136 | 1 | 1 | 1 | 6 | 3 |
| South Sudan | Fri, January 14, 2022 | 59 | 1 | 16,454 | 145 | 0 | 0 | 136 | 1 | 0 | -3 | -5 | -4 |
| Sudan | Fri, December 03, 2021 | 205 | 0 | 43,489 | 97 | 7 | 0 | 3,164 | 7 | 0 | 2 | 2 | 3 |
| Sudan | Fri, December 10, 2021 | 149 | 0 | 44,529 | 99 | 6 | 0 | 3,208 | 7 | 1 | -1 | -3 | -1 |
| Sudan | Fri, December 17, 2021 | 83 | 0 | 45,112 | 100 | 6 | 0 | 3,252 | 7 | 0 | -1 | -0 | -1 |
| Sudan | Fri, December 24, 2021 | 141 | 0 | 46,100 | 103 | 7 | 0 | 3,298 | 7 | 0 | 1 | 2 | 1 |
| Sudan | Fri, December 31, 2021 | 60 | 0 | 46,518 | 104 | 5 | 0 | 3,331 | 7 | 0 | -1 | -2 | -1 |
| Sudan | Fri, January 07, 2022 | 132 | 0 | 47,443 | 106 | 1 | 0 | 3,340 | 7 | 0 | 1 | 2 | 2 |
| Sudan | Fri, January 14, 2022 | 381 | 1 | 50,111 | 112 | 3 | 0 | 3,358 | 7 | 0 | 4 | 3 | 5 |
| Swaziland | Fri, December 03, 2021 | 91 | 8 | 47,158 | 4,022 | 0 | 0 | 1,248 | 106 | 0 | 51 | 49 | 52 |
| Swaziland | Fri, December 10, 2021 | 573 | 49 | 51,172 | 4,365 | 0 | 0 | 1,248 | 106 | 13 | 288 | 238 | 314 |
| Swaziland | Fri, December 17, 2021 | 1,101 | 94 | 58,876 | 5,022 | 1 | 0 | 1,258 | 107 | 57 | 315 | 26 | 455 |
| Swaziland | Fri, December 24, 2021 | 725 | 62 | 63,953 | 5,455 | 2 | 0 | 1,272 | 108 | 124 | -224 | -539 | -312 |
| Swaziland | Fri, December 31, 2021 | 308 | 26 | 66,109 | 5,639 | 4 | 0 | 1,303 | 111 | 82 | -249 | -25 | -214 |
| Swaziland | Fri, January 07, 2022 | 152 | 13 | 67,171 | 5,730 | 4 | 0 | 1,329 | 113 | 35 | -93 | 156 | -92 |
| Swaziland | Fri, January 14, 2022 | 87 | 7 | 67,777 | 5,781 | 4 | 0 | 1,356 | 116 | 6 | -39 | 54 | -45 |
| Tanzania | Fri, December 03, 2021 | 1 | 0 | 26,270 | 43 | 0 | 0 | 730 | 1 | 0 | -0 | -0 | -0 |
| Tanzania | Fri, December 10, 2021 | 6 | 0 | 26,309 | 43 | 1 | 0 | 734 | 1 | 0 | 0 | 0 | 0 |
| Tanzania | Fri, December 17, 2021 | 25 | 0 | 26,483 | 43 | 0 | 0 | 734 | 1 | 0 | 0 | 0 | 0 |
| Tanzania | Fri, December 24, 2021 | 0 | 0 | 26,483 | 43 | 0 | 0 | 734 | 1 | 0 | -0 | -1 | 0 |
| Tanzania | Fri, December 31, 2021 | 403 | 1 | 29,306 | 48 | 0 | 0 | 737 | 1 | 0 | 5 | 5 | 5 |
| Tanzania | Fri, January 07, 2022 | 180 | 0 | 30,564 | 50 | 0 | 0 | 740 | 1 | 1 | -3 | -7 | -2 |
| Tanzania | Fri, January 14, 2022 | 0 | 0 | 30,564 | 50 | 0 | 0 | 740 | 1 | 0 | -2 | 0 | 0 |
| Togo | Fri, December 03, 2021 | 7 | 0 | 26,284 | 310 | 0 | 0 | 243 | 3 | 0 | 0 | 0 | 0 |
| Togo | Fri, December 10, 2021 | 13 | 0 | 26,372 | 311 | 0 | 0 | 243 | 3 | 0 | 0 | 0 | 1 |
| Togo | Fri, December 17, 2021 | 25 | 0 | 26,550 | 313 | 0 | 0 | 244 | 3 | 0 | 1 | 1 | 1 |
| Togo | Fri, December 24, 2021 | 122 | 1 | 27,405 | 323 | 0 | 0 | 246 | 3 | 0 | 8 | 7 | 9 |
| Togo | Fri, December 31, 2021 | 394 | 5 | 30,163 | 356 | 0 | 0 | 248 | 3 | 2 | 22 | 14 | 27 |
| Togo | Fri, January 07, 2022 | 484 | 6 | 33,553 | 396 | 1 | 0 | 252 | 3 | 6 | 7 | -15 | 17 |
| Togo | Fri, January 14, 2022 | 302 | 4 | 35,665 | 421 | 1 | 0 | 261 | 3 | 3 | -15 | -23 | -19 |
| Uganda | Fri, December 03, 2021 | 34 | 0 | 127,651 | 271 | 0 | 0 | 3,253 | 7 | 0 | -0 | -0 | -0 |
| Uganda | Fri, December 10, 2021 | 42 | 0 | 127,944 | 272 | 2 | 0 | 3,265 | 7 | 0 | 0 | 0 | 0 |
| Uganda | Fri, December 17, 2021 | 91 | 0 | 128,578 | 273 | 1 | 0 | 3,272 | 7 | 0 | 1 | 1 | 1 |
| Uganda | Fri, December 24, 2021 | 486 | 1 | 131,981 | 280 | 0 | 0 | 3,274 | 7 | 0 | 6 | 5 | 7 |
| Uganda | Fri, December 31, 2021 | 1,251 | 3 | 140,737 | 299 | 3 | 0 | 3,294 | 7 | 1 | 11 | 5 | 15 |
| Uganda | Fri, January 07, 2022 | 1,376 | 3 | 150,368 | 319 | 4 | 0 | 3,325 | 7 | 4 | 2 | -10 | 6 |
| Uganda | Fri, January 14, 2022 | 980 | 2 | 157,227 | 334 | 9 | 0 | 3,385 | 7 | 1 | -6 | -8 | -9 |
| Zambia | Fri, December 03, 2021 | 21 | 0 | 210,262 | 1,111 | 0 | 0 | 3,667 | 19 | 0 | 0 | 0 | 1 |
| Zambia | Fri, December 10, 2021 | 66 | 0 | 210,724 | 1,114 | 0 | 0 | 3,668 | 19 | 0 | 2 | 1 | 2 |
| Zambia | Fri, December 17, 2021 | 530 | 3 | 214,433 | 1,133 | 1 | 0 | 3,674 | 19 | 0 | 17 | 15 | 18 |
| Zambia | Fri, December 24, 2021 | 2,071 | 11 | 228,932 | 1,210 | 2 | 0 | 3,691 | 20 | 4 | 57 | 40 | 66 |
| Zambia | Fri, December 31, 2021 | 3,620 | 19 | 254,274 | 1,344 | 6 | 0 | 3,734 | 20 | 15 | 57 | 0 | 88 |
| Zambia | Fri, January 07, 2022 | 3,429 | 18 | 278,276 | 1,471 | 8 | 0 | 3,790 | 20 | 26 | -7 | -64 | -30 |
| Zambia | Fri, January 14, 2022 | 2,203 | 12 | 293,695 | 1,552 | 8 | 0 | 3,844 | 20 | 9 | -45 | -38 | -61 |
| Zimbabwe | Fri, December 03, 2021 | 515 | 3 | 137,441 | 911 | 1 | 0 | 4,708 | 31 | 0 | 22 | 22 | 23 |
| Zimbabwe | Fri, December 10, 2021 | 2,625 | 17 | 155,817 | 1,032 | 2 | 0 | 4,723 | 31 | 6 | 98 | 76 | 109 |
| Zimbabwe | Fri, December 17, 2021 | 4,821 | 32 | 189,567 | 1,256 | 8 | 0 | 4,779 | 32 | 20 | 102 | 4 | 151 |
| Zimbabwe | Fri, December 24, 2021 | 1,881 | 12 | 202,736 | 1,343 | 13 | 0 | 4,871 | 32 | 42 | -136 | -238 | -109 |
| Zimbabwe | Fri, December 31, 2021 | 1,503 | 10 | 213,258 | 1,413 | 19 | 0 | 5,004 | 33 | 17 | -18 | 119 | -35 |
| Zimbabwe | Fri, January 07, 2022 | 1,146 | 8 | 221,282 | 1,466 | 19 | 0 | 5,136 | 34 | 13 | -17 | 1 | -30 |
| Zimbabwe | Fri, January 14, 2022 | 622 | 4 | 225,637 | 1,495 | 15 | 0 | 5,238 | 35 | 4 | -24 | -8 | -26 |
